# The parathyroid hormone regulates skin tumour susceptibility in mice

**DOI:** 10.1038/s41598-017-11561-x

**Published:** 2017-09-11

**Authors:** Kazuhiro Okumura, Megumi Saito, Yasuhiro Yoshizawa, Haruka Munakata, Eriko Isogai, Ikuo Miura, Shigeharu Wakana, Midori Yamaguchi, Hiroshi Shitara, Choji Taya, Andrew C. Karaplis, Ryo Kominami, Yuichi Wakabayashi

**Affiliations:** 1 Department of Carcinogenesis Research, Division of Experimental Animal Research, Chiba Cancer Center Research Institute, 666-2 Nitonacho Chuouku, Chiba, 260-8717 Japan; 2 Technology and Development Team for Mouse Phenotype Analysis: Japan Mouse Clinic, Riken Bioresource Center, 3-1-1 Koyadai, Tsukuba, Ibaraki 305-0074 Japan; 3 Laboratory for Transgenic Technology, Tokyo Metropolitan Institute of Medical Science, 1-6, Kamikitazawa 2-chome, Setagaya-ku, Tokyo, 156-8506 Japan; 4 McGill University and Jewish General Hospital, 3755 Côte-Ste-Catherine Road, E-104 Montreal, Quebec, H3T 1E2 Canada; 5 Department of Molecular Physiology, Niigata University School of Medicine, Asahimachi 1-757, Niigata, 951-8510 Japan

## Abstract

Using a forward genetics approach to map loci in a mouse skin cancer model, we previously identified a genetic locus, *Skin tumour modifier of MSM* 1 (*Stmm*1) on chromosome 7, conferring strong tumour resistance. Sub-congenic mapping localized *Parathyroid hormone* (*Pth*) in *Stmm1b*. Here, we report that serum intact-PTH (iPTH) and a genetic polymorphism in *Pth* are important for skin tumour resistance. We identified higher iPTH levels in sera from cancer-resistant MSM/Ms mice compared with susceptible FVB/NJ mice. Therefore, we performed skin carcinogenesis experiments with MSM-BAC transgenic mice (*Pth* ^MSM^-*Tg*) and *Pth* knockout heterozygous mice (*Pth* ^+/−^). As a result, the higher amounts of iPTH in sera conferred stronger resistance to skin tumours. Furthermore, we found that the coding SNP (rs51104087, Val28Met) localizes in the mouse Pro-PTH encoding region, which is linked to processing efficacy and increased PTH secretion. Finally, we report that PTH increases intracellular calcium in keratinocytes and promotes their terminal differentiation. Taken together, our data suggest that *Pth* is one of the genes responsible for *Stmm1*, and serum iPTH could serve as a prevention marker of skin cancer and a target for new therapies.

## Introduction

The risk of developing cancer is regulated by a combination of, and interactions between, genetic and environmental factors. Although novel environmental risks will continue to be discovered, many of the major cancer-causing environmental reagents have been recognized and have become the focus of strategies toward prevention. The role of genetics in cancer risk has been well established with the discovery of mutations in high penetrance genes that segregate in families. However, the contribution of high penetrance genes to the overall human cancer burden is small because of their low frequencies in human populations^1^. Instead, the majority of cancer risk has been proposed to be regulated by commonly occurring low penetrance genetic variants, and this has led to a flurry of association studies to compare the frequency of specific variants between cancer patients and normal controls. These studies involve hundreds to tens of thousands of individuals, and yet they continue to be plagued by factors that are difficult to control for such as population heterogeneity, environmental exposure, and weak effects of variants^2–4^. A number of low penetrance human cancer susceptibility alleles have indeed been identified and validated using this approach, but their roles in cancer risk are not fully understood because the effects of their interactions with one another or with other variants have not been well elucidated^1^.

Studies in mice overcome many of the limiting factors of human association studies, and are therefore an important alternative approach to the study of cancer susceptibility. Studies on mice have revealed that tumour predisposition in different strains is controlled by multiple loci that exhibit complex genetic interactions, and these strains could be inter-bred to generate genetically heterogeneous offspring to recapitulate human populations that could be maintained under controlled environment^5, 6^. Importantly, in these mouse crosses, only two alleles at each locus are segregating in the population, and the differences in susceptibility are therefore due to combinations of common alleles. Studies involving genetic crosses between cancer resistant and susceptible strains of mice have led to the identification of a number of genes with important roles in cancer susceptibility and cancer biology, some of which have clear translatability to human cancer^7–16^.

Previously, we reported that the Japanese wild-derived mouse strain MSM/Ms (MSM) is dominantly resistant to chemically induced skin tumour development^17^ and genetic crosses with the cancer susceptible FVB/N (FVB) strain of mice have led to the identification of genetic loci with strong effects on cancer risk. We focused on *Skin tumour modifier of MSM 1* (*Stmm1*) on chromosome 7, and have generated sub-congenic lines to refine the locus and identify the gene responsible for the effect of the locus^17, 18^. Herein, we identify *parathyroid hormone* (*Pth*) as the major candidate for *Stmm1*. PTH is well known to act with vitamin D to regulate calcium and phosphate homeostasis^19–21^. Currently, it is well known that skin acts as neuroendocrine organ. Almost all the elements controlling the activity of the hypothalamus-pituitary-adrenal axis are expressed in the skin as previously described in detail^22^. PTH and PTH related peptide (PTHrP) also influence the proliferation and differentiation of epidermal cells^23–26^ through paracrine and intracrine routes^27^, but the role of PTH in skin carcinogenesis is poorly understood. We found higher intact-PTH (iPTH) levels in sera from cancer resistant MSM/Ms mice compared with in susceptible FVB/N mice. These differences in sera iPTH levels were observed in MSM-BAC transgenic (*Pth* ^MSM^-*Tg*) mice, and were associated with their resistance to skin carcinogenesis. The genetic variant in PTH between MSM (i.e. Methionine) and FVB/N (i.e. Valine) occurs at the Pro-PTH region (amino acid position 28), and we demonstrate using *in vitro* studies that this change influences PTH peptide processing efficacy, with the *Pth* ^MSM^ allele having stronger acceleratory intracellular calcium levels, terminal differentiation and inhibitory effects on cell proliferation.

## Results

### Refined analysis of *Stmm1* locus by sub-congenic mapping

**Figure 1:**
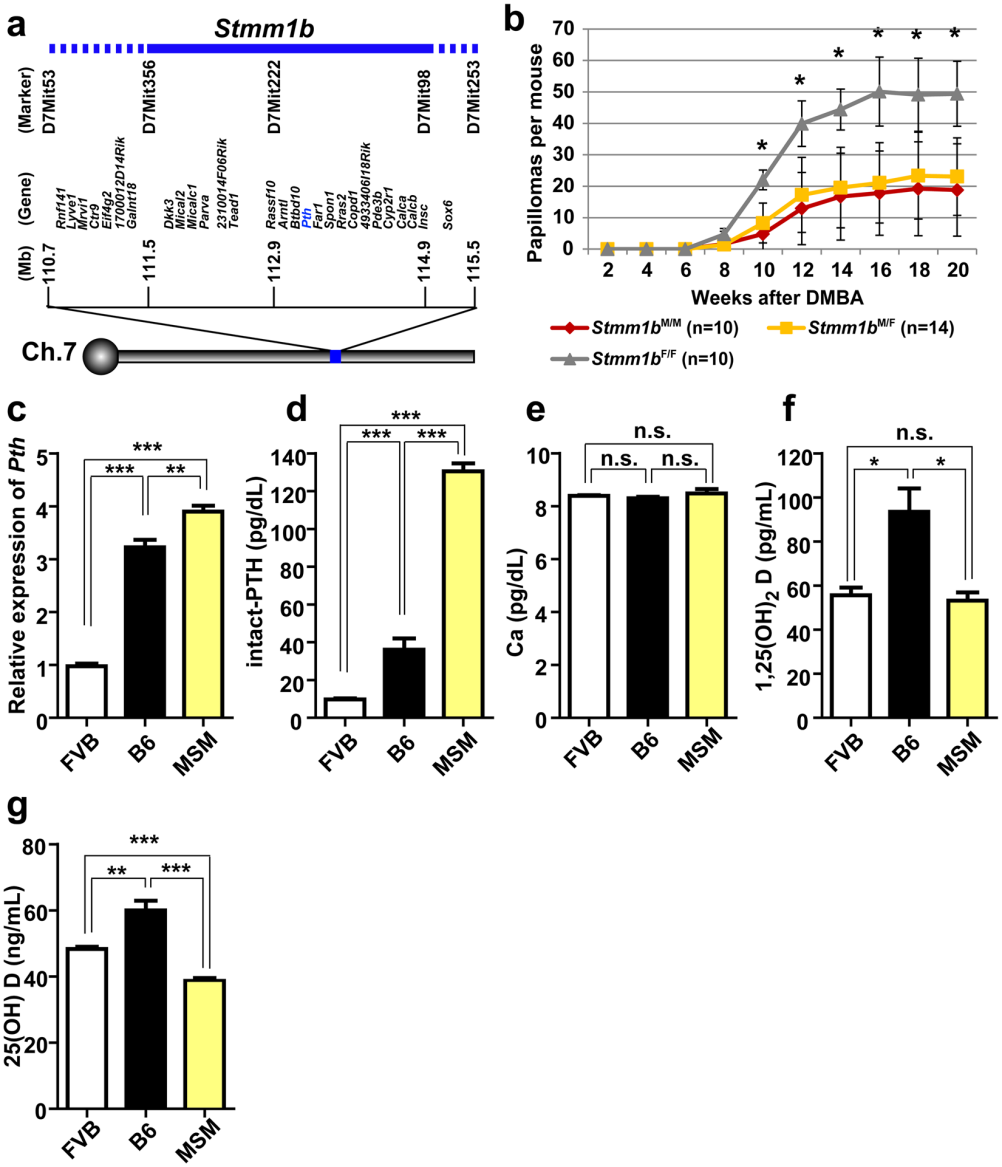
Parathyroid hormone (*Pth*) is located in the middle of the Skin Tumour Modifier of MSM 1b (*Stmm1b*) locus and serum intact-PTH is elevated in tumour resistant strains. (**a**) A schematic representation of the genetic position around *Stmm1b*. A blue bar represents the region of *Stmm1b*. Genetic positions shown are according to the Mouse Genome Informatics Database (MGI) (http://www.informatics.jax.org/). (**b**) Comparisons of the average papilloma number per mouse between heterozygous congenic line (*Stmm1b* ^M/F^) and homozygous congenic mice (*Stmm1b* ^M/M^ and *Stmm1b* ^F/F^). The *P*-values were calculated for papilloma number at 8–20 weeks by *t*-test (*P < 0.01). (**c**) The means of relative expression of *Pth* in whole P0 pups of FVB/N, C57BL6/J and MSM/Ms, respectively (n = 3) by qRT-PCR analysis. The *P*-values were calculated by *t*-test (***P < 0.001). (**d**) The means of serum intact-PTH concentrations in FVB/N, C57BL6/J and MSM/Ms, respectively (n = 4 each; female, 3 M) by mouse intact-PTH ELISA. The *P*-values were calculated by *t*-test (***P < 0.001). (**e**) The means of serum calcium ion (Ca^2+^) concentrations in FVB/N, C57BL6/J and MSM/Ms by o-Cresolphthalein-complexone (oCPC) method (n = 4 each; female, 3 M). n.s., no significant differences. (**f**) The means of serum 1,25(OH)_2_ vitamin D concentrations in FVB/N, MSM/Ms and C57B6/J by the Radio Immuno Assay (RIA) (n = 3 each; female, 3 M). (**g**) The means of serum 25(OH) vitamin D concentrations in FVB/N, MSM/Ms and C57B6/J by the Radio Immuno Assay (RIA) (n = 3 each; female, 2 M). The *P*-values were calculated by *t*-test (*P < 0.05). n.s., no significant differences. (**b**–**g**) Error bars represent the standard deviation (S.D.). Asterisks indicate significant differences.

We have recently identified a skin papilloma resistant locus, *Stmm1* (*S* *kin* *t* *umour modifier of* *M* *SM* *1*) locus on chromosome 7, using F_1_ backcross mice between a wild derived inbred mouse strain (MSM/Ms) and a susceptible inbred mouse strain (FVB/N)^17^. Previous QTL analysis with a different wild derived inbred mouse strain (*Mus*. *Spret*) also identified skin tumour susceptibility loci (*Skts1* and *Skts2*) on mouse chromosome 7^7^. Using a panel of congenic mouse strains, we refined the *Stmm1* locus to within a genetic interval of approximately 3 cM on chromosome 7^18^. *Stmm1* confers resistance to early-mid stage papilloma development by chemical two-stage skin carcinogenesis (DMBA/TPA). To further refine the *Stmm1* locus for candidate gene identification, we generated a sub-congenic mouse line (*Stmm1b*) that spans D7Mit356 (111,514,329-111,514,441 bp) to D7Mit98 (114,917,267-114,917,439 bp) (Fig. 1a). In skin carcinogenesis experiments, *Stmm1b*-homozygous MSM/MSM (*Stmm1b* ^M/M^) mice (n = 10) were the most resistant to papilloma development (18.8 ± 14.7 papillomas at 20 weeks after initiation), while homozygous FVB/FVB (*Stmm1b* ^F/F^) mice (n = 10) were the most susceptible (49.4 ± 10.3). *Stmm1b*-heterozygous MSM/FVB (*Stmm1b* ^M/F^) mice (n = 14) were intermediate, having developed 23.0 ± 12.3 papillomas (Fig. 1b). Therefore, *Stmm1b* was narrowed down to an approximately 3.4 Mb region between D7Mit356 (58.21 cM) and D7Mit98 (60.49 cM) by sub-congenic mapping analysis. These differences were significant, indicating that the gene responsible for the effects of *Stmm* is contained within the *Stmm1b* congenic region.

### The assessment of *Pth* expression

Among the genes located within the *Stmm1b* region is *Pth*, which previous studies have reported affects proliferation and differentiation of epidermal cells, suggesting that it may play a role in skin carcinogenesis^23–25^. However, the function of PTH in skin is poorly understood. Therefore, we investigated the expression of *Pth* in skin from FVB, B6 and MSM strains. Based on *in situ* hybridization, *Pth* expression was observed in parathyroid tissue of whole 18.5 embryos but not in adult skin tissues in FVB and MSM mice (Supplementary Fig. 1a). qRT-PCR to measure *Pth* transcripts in whole P0 pups showed significant expression changes between FVB and MSM mice (Fig. 1c). Next, we performed analysis of PTH protein expression levels in sera from FVB, MSM and also B6 mice as a control by specific intact-PTH (iPTH) ELISA assay. As a result, the amounts of iPTH were ~10.0-fold higher in MSM than in FVB (Fig. 1d). In contrast, we did not detect a significant difference in the serum calcium (Ca) or dihydroxy vitamin D (1,25(OH)_2_D) concentrations between FVB and MSM (Fig. 1e,f). In addition, we detected a small difference in 25(OH) D concentration between FVB and MSM (Fig. 1g). We also checked the amounts of Ca and iPTH in sera from *Stmm1* ^M/F^ and *Stmm1* ^F/F^ congenic lines, which exhibited no significant differences in the Ca concentration (Supplementary Fig. 1b). However, the amounts of iPTH were ~2.0-fold higher in *Stmm1* ^M/F^ than in *Stmm1* ^F/F^ mice (Supplementary Fig. 1c). PTH action is mediated primarily through the binding and activation of the PTH receptor type 1 (PTH1R), which is highly expressed in PTH target tissues^28^. In addition to PTH, PTH1R also recognizes PTHrP, a paracrine/autocrine factor originally discovered in many tumours that causes the syndrome of malignancy-associated hypercalcemia^29, 30^. We then compared *Pthrp* and *Pth1r* transcripts in the adult skin among FVB, B6 and MSM. We did not detect a significant difference in the expression of *Pthrp* and *Pth1r* between FVB and MSM (Supplementary Fig. 2a,b).

### *Pth*^MSM^ allele confers resistance to two-stage skin carcinogenesis

**Figure 2:**
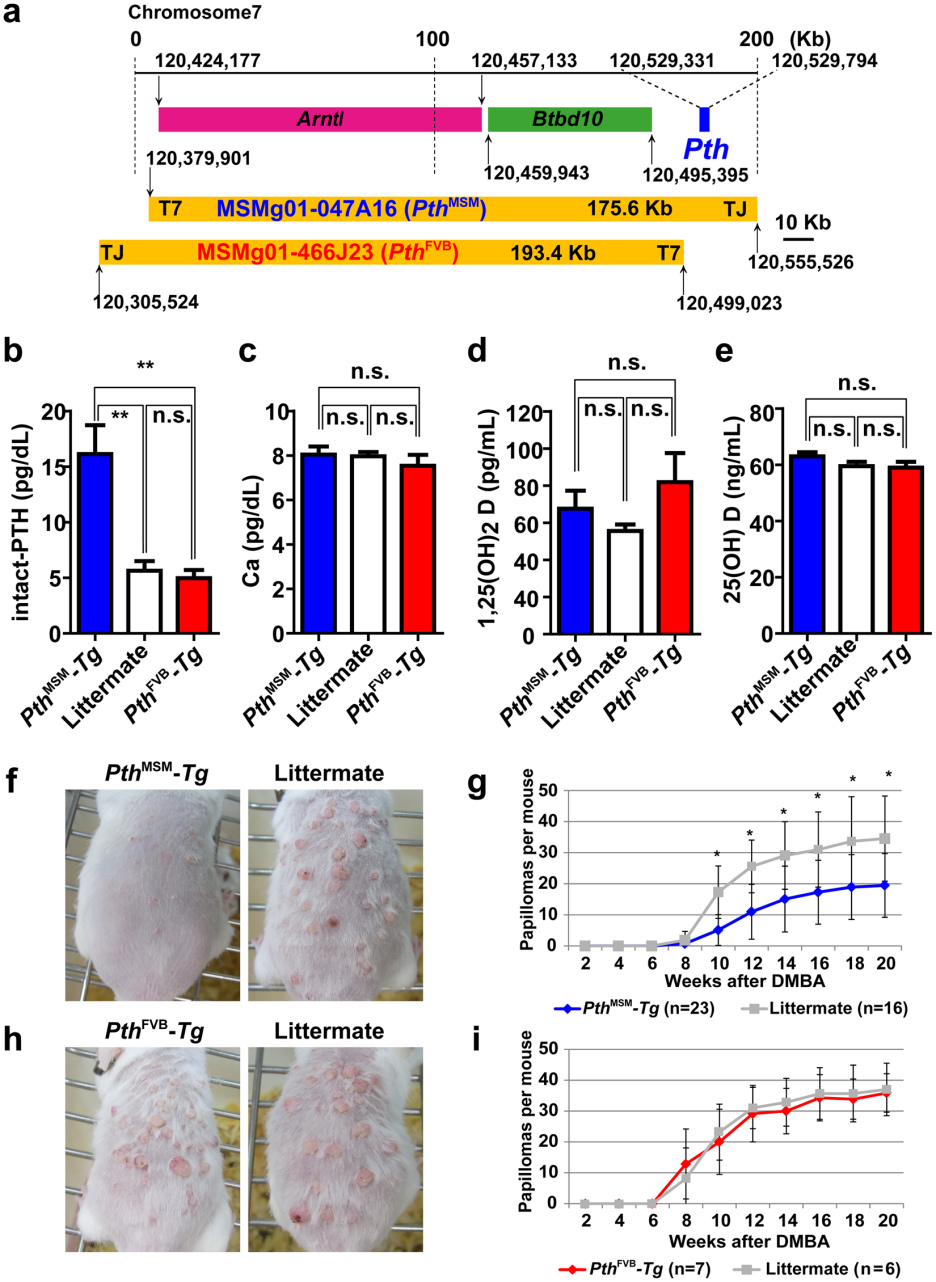
*Pth* ^MSM^ allele confers resistance to skin tumours, which is confirmed using MSM-BAC transgenic mice (*Pth* ^MSM^-*Tg*). (**a**) A schematic drawing of the genetic location of two MSM-BAC clones (MSMg01-047A16 and MSMg01-466J23). Shown genetic positions are according to the NIG Mouse genome database (http://molossinus.lab.nig.ac.jp/msmdb/index.jsp). MSM-BAC clones were microinjected into the pronuclei and cytoplasm of fertilized FVB mouse eggs (see Material and Methods). (**b**) The means of serum intact-PTH concentrations in *Pth* ^MSM^-*Tg*, littermate mice and *Pth* ^FVB^-*Tg* (n = 4 each; female, 3 M) by ELISA. (**c**) The means of serum calcium ion (Ca^2+^) concentrations in *Pth* ^MSM^-*Tg*, littermate mice and *Pth* ^FVB^-*Tg* (n = 4 each; female, 3 M) by oCPC method. n.s., no significant differences. (**d**) The means of concentrations of the serum 1,25(OH)_2_ vitamin D in *Pth* ^MSM^-*Tg*, littermate mice and *Pth* ^FVB^-*Tg* (n = 4 each; female, 3 M) by RIA. The *P*-values were calculated by *t*-test (**P < 0.01). n.s., no significant differences. (**e**) The means of concentrations of the serum 25(OH) vitamin D in *Pth* ^MSM^-*Tg*, littermate mice and *Pth* ^FVB^-*Tg* (n = 3 each; female, 3 M) by RIA. The *P*-values were calculated by *t*-test (**P < 0.01). n.s., no significant differences. (**f**) Dorsal back skin of *Pth* ^MSM^-*Tg* and littermate mice at 12 weeks after DMBA initiation. (**g**) Comparison of the average papilloma number per mouse between *Pth* ^MSM^-*Tg* and littermate mice. (**h**) Dorsal back skin of *Pth* ^FVB^-*Tg* and littermate mice at 12 weeks after DMBA initiation. (**i**) Comparison of the average papilloma number per mouse between *Pth* ^FVB^-*Tg* and littermate mice. The *P*-values were calculated for papilloma number at 10–20 weeks by *t*-test (*P < 0.01). (**b**–**i**) Error bars represent the standard deviation (S.D.). (**d**,**g**) Asterisks indicate significant differences.

To investigate whether *Pth* ^MSM^ allele confers resistance to skin carcinogenesis or not, we carried out BAC transgenesis experiments using BAC clones from RIKEN MSM BAC clone library. MSMg01-047A16 covered an approximately 20 kb region upstream of *Pth*, including *Btbd10* and *Arntl* genes (Fig. 2a). In contrast, MSMg01-466J23 excluded *Pth*, and instead included *Btbd10* and *Arntl* genes (Fig. 2a). These clones were injected into FVB/N eggs, respectively. Therefore, the genetic backgrounds of *Tg(047A16)* (*Pth* ^MSM^ *-Tg*) and *Tg(466J23)* (*Pth* ^FVB^ *-Tg*) mouse lines were FVB/N mice except transgenes. We first checked the transgene expression by allele-specific PCR using F_1_ hybrid (FVB × MSM) as a control for one copy of each allele. As a result, we observed that *Pth* ^MSM^ *-Tg* had 4 copies of the *Pth* ^MSM^ allele and 2 copies of the *Pth* ^FVB^ allele. Conversely, *Pth* ^FVB^ *-Tg* had only 2 copies of the *Pth* ^FVB^ allele (Supplementary Fig. 3a). Moreover, we checked the total amounts of *Pth* mRNA expression in *Tg* mice. qRT-PCR analysis revealed that *Pth* ^MSM^ *-Tg* mice showed equivalent *Pth* mRNA expression levels when compared with F_1_ and MSM (Supplementary Fig. 3b). We further measured the amounts of serum iPTH in *Pth* ^MSM^ *-Tg* and *Pth* ^FVB^ *-Tg* mice. As a result, the concentration of serum iPTH was two-times higher in *Pth* ^MSM^ *-Tg* than in *Pth* ^FVB^ *-Tg* mice (Fig. 2b). In contrast, we did not detect significant differences in serum Ca or 1,25(OH)_2_D and 25(OH) D concentrations (Fig. 2c–e). Taken together, we successfully generated FVB-*Pth* ^MSM^ *-Tg* mice with a high serum iPTH concentration. To determine the role of the *Pth* ^MSM^ allele, *Pth-Tg* mice were subjected to the DMBA-TPA skin carcinogenesis experiment (Fig. 2f–i). We monitored development of papillomas in these mice for 20 weeks after DMBA treatment. As a result, *Pth* ^MSM^ *-Tg* (n = 23) developed an average of 5.0 ± 4.9 and 20.5 ± 9.51 papillomas/mouse at 10 and 20 weeks after initiation, respectively (*P*-value from *t*-test) (Fig. 2f,g). In contrast, littermate mice (n = 16) were highly susceptible to skin tumourigenesis. They developed on average 17.3 ± 8.4 papillomas at 10 weeks after initiation and 34.5 ± 13.6 papillomas at 20 weeks after initiation (Fig. 2f,g). On the other hand, *Pth* ^FVB^ *-Tg* (n = 7) developed on average 35.8 ± 6.2 papillomas at 20 weeks after initiation, which was similar with littermate (n = 6) mice (Fig. 2h,i). For further validation, we also carried out the skin carcinogenesis experiment with another *Tg* mice line (*Tg(7-13)*), which was generated by differentially injecting MSMg01-047A16 clone including *Pth* ^MSM^. As a result, *Tg(7-13)* mice (n = 10) exhibited a similar number of papillomas with *Pth* ^MSM^ *-Tg* mice at 20 weeks after initiation (Supplementary Fig. 3c).

### *Pth*^+/−^ heterozygous mice are susceptible to two-stage skin carcinogenesis

**Figure 3:**
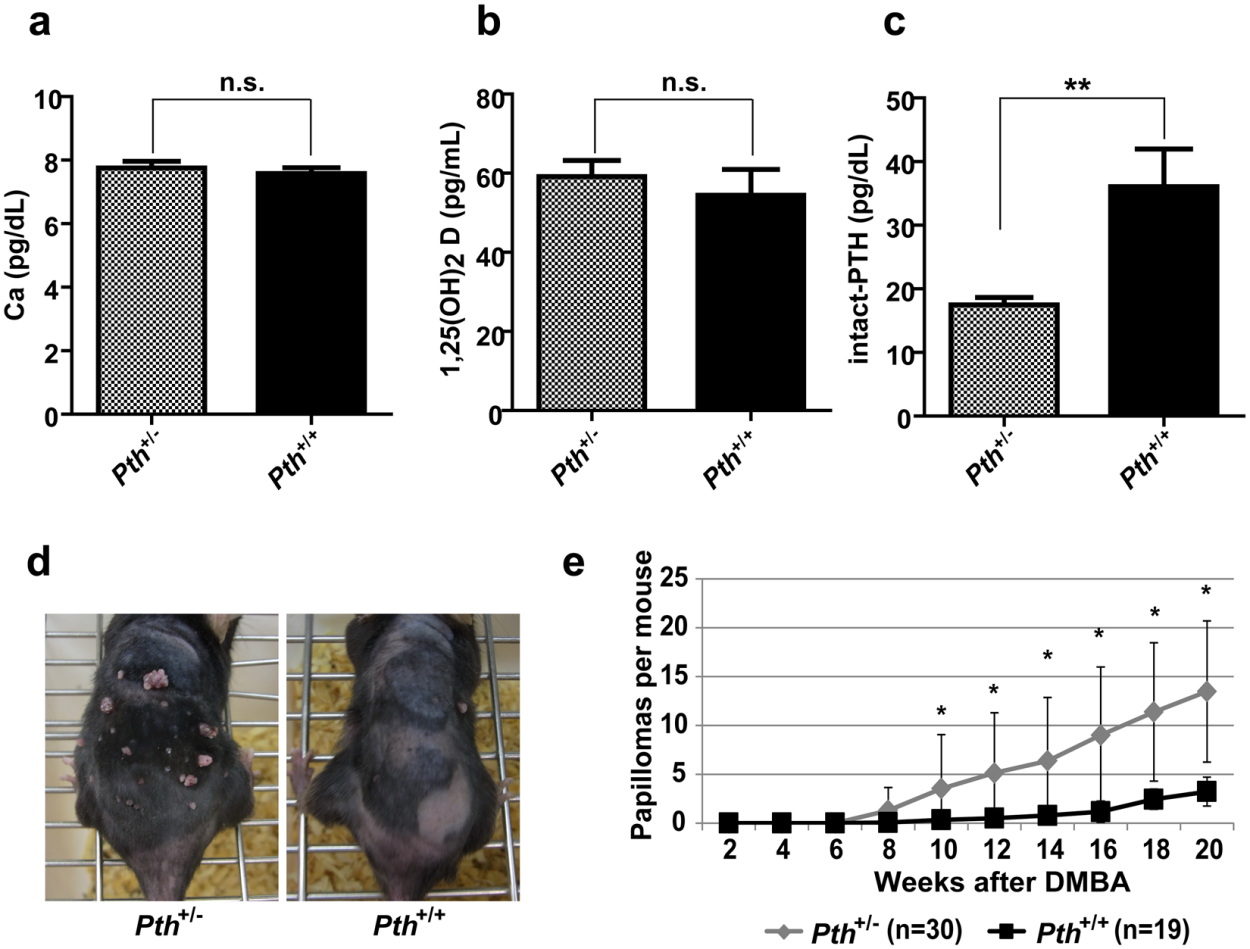
*Pth* ^+/−^ knock-out heterozygous mice are susceptible to chemically induced skin carcinogenesis. (**a**) The mean of serum Ca^2+^ concentrations in heterozygous KO (*Pth* ^+/−^) and wild type (*Pth* ^+/+^) mice (n = 4 each; female, 3 M) by oCPC method. n.s., no significant differences. The *P*-values were calculated by *t*-test (**P < 0.01). (**b**) The mean of serum 1,25(OH)_2_ vitamin D concentrations in *Pth* ^−/−^, *Pth* ^+/−^ and *Pth* ^+/+^ mice (n = 3) by RIA. n.s., no significant differences. The *P*-values were calculated by *t*-test (***P < 0.001, **P < 0.01). (**c**) The means of serum intact-PTH concentrations in *Pth* ^+/−^ (n = 6) and *Pth* ^+/+^ (n = 3) mice by ELISA. The *P*-values were calculated by *t*-test (**P < 0.01). (**d**) Representative photographs of mice during the treatment with DMBA/TPA. *Pth* ^+/−^ (left) and *Pth* ^+/+^ mice (right) at 16 weeks after initiation. (**e**) Comparison of the average papilloma number per mouse between *Pth* ^+/−^ (n = 30) and *Pth* ^+/+^ mice (n = 19). The *P*-values were calculated for papilloma number at 8–20 weeks by *t*-test (*P < 0.01). (**a–c**,**e**) Error bars are the standard deviations (S.D.). Asterisks indicate significant differences.

We found higher iPTH levels in sera from cancer-resistant MSM/Ms mice compared with in susceptible FVB/N mice. To elucidate the mechanisms responsible for PTH-mediated tumour resistance, we also conducted skin carcinogenesis experiments with *Pth* knockout (*Pth* ^−/−^) mice. First, we measured Ca, 1,25(OH)_2_D and iPTH in sera from *Pth* ^+/−^ and *Pth* ^+/+^ mice. Previous studies have reported that *Pth* ^−/−^ mice demonstrated abnormal development^19^. In this study, *Pth* ^+/−^ mice exhibited similar Ca and vitamin D concentrations when compared with *Pth* ^+/+^ mice (Fig. 3a,b). In addition, iPTH concentration in sera was lower in *Pth* ^+/−^ than in *Pth* ^+/+^ mice (Fig. 3c). We used *Pth* ^+/−^ and *Pth* ^+/+^ mice for the two-stage skin carcinogenesis experiment because we could exclude the possibility that differences in Ca and 1,25(OH)_2_D concentrations in sera have an influence on skin carcinogenesis experiments^31^. In Fig. 3d, the photograph shows dorsal back skin of *Pth* ^+/−^ and *Pth* ^+/+^ mice at 16 weeks after DMBA treatment. We monitored the development of papillomas in *Pth* ^+/−^ and *Pth* ^+/+^ mice for 20 weeks after the DMBA treatment period (Fig. 3e). As a result, *Pth* ^+/−^ mice (n = 30) were highly susceptible to skin carcinogenesis, and developed on average 13.4 ± 7.2 papillomas at 20 weeks after initiation (Fig. 3e). In contrast, *Pth* ^+/+^ (n = 19) developed an average of 3.2 ± 1.4 papillomas/mouse at 20 weeks after initiation (Fig. 3e). Therefore, *Pth* ^+/−^ mice were susceptible to chemically induced skin tumours when compared with *Pth* ^+/+^ mice. These results indicate that *Pth* haploinsufficiency impacts skin carcinogenesis and high iPTH concentration in sera led to the decrease of papilloma number in mice.

### The amounts of secreted PTH-GFP are elevated in *Pth*^MSM^-transfected NIH-3T3 cells

**Figure 4:**
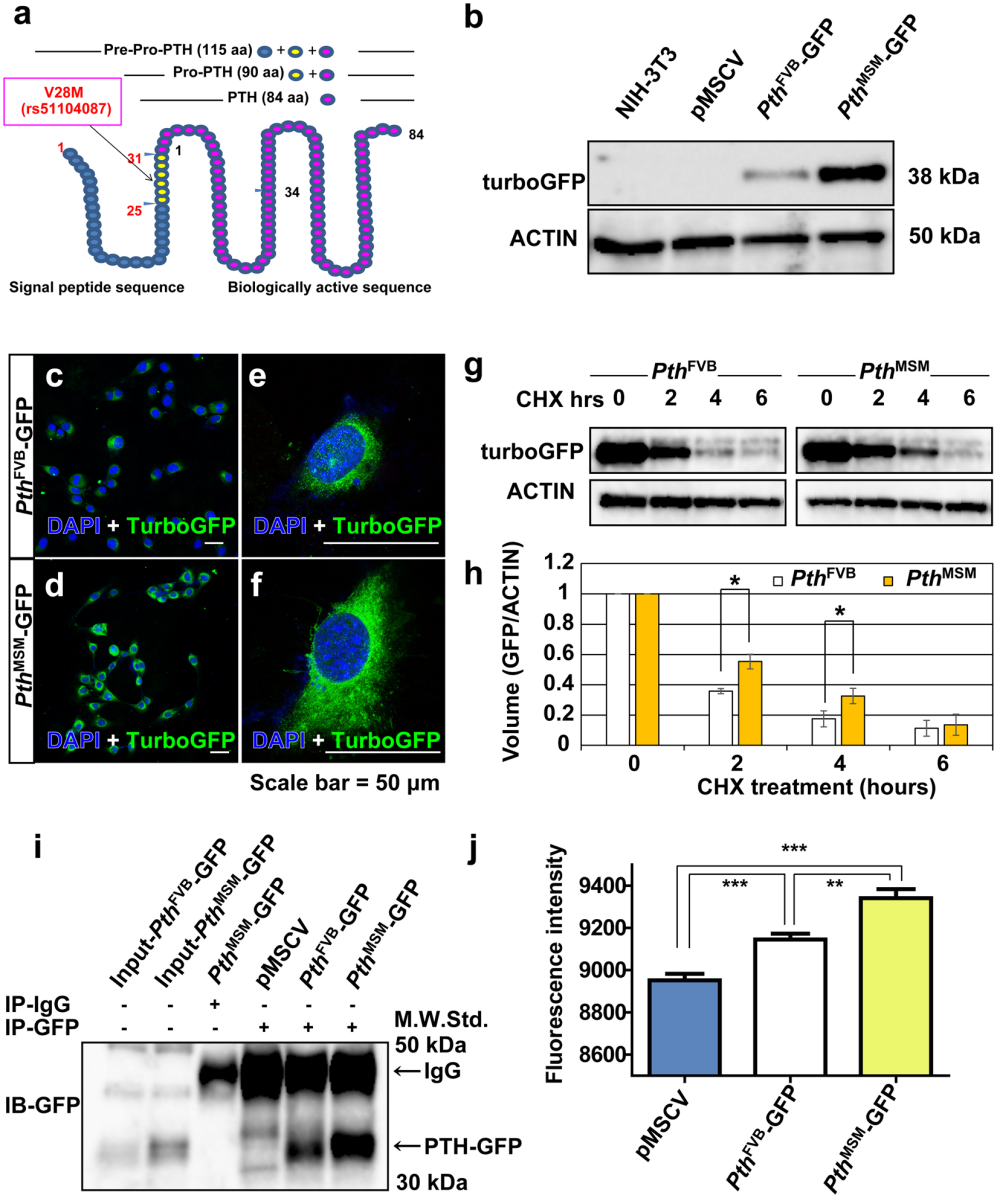
A non-synonymous substitution (V28M) in Pro-PTH effects the amounts of intracellular and secreted PTH-GFP. (**a**) This figure shows the full-length sequence of PTH. PTH is synthesized as a pre-pro-hormone. Blue circles indicate signal peptide sequence. Yellow circles indicate Pro-PTH region. Pink circles indicate the biologically active sequence. A SNP (Val28Met) is located in the Pro-PTH region. (**b**) The protein expression pattern of PTH-GFP in *Pth* ^FVB^ and *Pth* ^MSM^-transfected NIH-3T3 cells. Proteins isolated from *Pth* ^FVB^- and *Pth* ^MSM^-transfected NIH-3T3 cells were subjected to immunoblot analysis with anti-turboGFP antibody. The amounts of protein loaded per well were confirmed by ACTIN. (**c**–**f**) Immunofluorescence staining of turboGFP in *Pth* ^FVB^ (**c**,**e**)- and *Pth* ^MSM^ (**d**,**f**)-transfected NIH-3T3 cells. (**e**) A magnified image of (**c**). (**f**) A magnified image of (**d**). Transfected cells were stained with anti-turboGFP antibody (green). Cells were counterstained with DAPI (blue). Scale bars, 50 μm. (**g**) *Pth* ^FVB^- and *Pth* ^MSM^-transfected NIH-3T3 cells were treated with 100 μg/ml of cycloheximide (Chx) for 6 hours. Cells were lysed at the indicated time points. Cell lysates were subjected to immunoblot analysis with anti-turboGFP and anti-ACTIN antibodies. ACTIN was used as a control for equal protein loading and integrity. Blots were cropped from different parts of the same blot. The full-length blots are shown in Supplementary Fig. 9. (**h**) Comparison of the amounts PTH-GFP between *Pth* ^FVB^- and *Pth* ^MSM^-transfected NIH-3T3 cells. The *P*-value was calculated by *t*-test for band intensity (*P < 0.05). (**i**) Detection of secreted PTH-GFP by immunoprecipitation and immunoblot. Lanes 1 and 2 indicate input serum-free culture medium from *Pth* ^FVB^- and *Pth* ^MSM^-transfected NIH-3T3 cells. Lane 3 indicates serum-free culture medium of *Pth* ^MSM^ immunoprecipitated using IgG. Lane 4 indicates serum-free culture medium of control vector immunoprecipitated using anti-turboGFP. Lane 5 and 6 indicate serum-free culture medium of *Pth* ^FVB^ and *Pth* ^MSM^ immunoprecipitated using anti-turboGFP. The images with three different exposure times are shown in Supplementary Fig. 10. (**j**) Basic GFP fluorescent intensity of serum-free culture medium of pMSCV (control) (n = 3), *Pth* ^FVB^ (n = 3)- and *Pth* ^MSM^ (n = 3)-transfected NIH3T3 cells using microplate readers. The *P*-values were calculated by *t*-test (***P < 0.001, **P < 0.01). (**h**,**j**) Error bars are standard deviation (SD). Asterisks indicate significant differences.

We next examined the genomic and amino acid sequence of *Pth* in different mammalian species using several databases and found non-synonymous substitution (V28M: amino acid 28 changed from Valine to Methionine) in MSM (Supplementary Fig. 4a). PTH is synthesized as a pre-pro-hormone in parathyroid cells and processed to mature PTH comprising 84 amino acids (Fig. 4a). PTH1-34 is biologically an active agonist and PTH1-6 is important for binding with PTH1R. We focused on the non-synonymous substitution, which was close to the processing site of the Pro-PTH form. To investigate the function of the genetic polymorphism in *Pth* (Val28Met: rs51104087), we generated the construct vectors for recombinant Pre-Pro (Val)-1-84-turboGFP (*Pth* ^FVB^-GFP) and Pre-Pro (Met)-1-84-turboGFP (*Pth* ^MSM^-GFP) (Supplementary Fig. 4b). These vectors were then transduced into NIH-3T3 cells by retroviral infection system and induction of recombinant protein was checked by immunoblotting (IB) (data not shown). As established mouse parathyroid cell lines are not available, we chose the NIH-3T3 cell line as a cell line having protein secretory capacity like parathyroid cells^32^. Cells were selected with puromycin for four weeks and expression levels of *Pth* mRNA in individual clones were determined. Although similar expression levels of *Pth* mRNA were observed (Supplementary Fig. 4c), the amounts of intracellular PTH fusion GFP protein were higher in *Pth* ^MSM^ than in *Pth* ^FVB^ by IB analysis (Fig. 4b). Next, we investigated the intracellular localization of PTH fusion GFP by immunostaining for PTH^FVB^ and PTH^MSM^ in the cells. As a result, the amounts of PTH fusion GFP protein were higher in *Pth* ^MSM^ than in *Pth* ^FVB^. PTH^MSM^ was widespread in the cells (Fig. 4c–f). The main localization of both alleles of PTH fusion GFP was the Golgi and the endoplasmic reticulum (ER) (Supplementary Fig. 5a–d). In addition, the majority of *Pth* ^MSM^-GFP was detected in the ER (Supplementary Fig. 5c,d). Therefore, we examined the effects of brefeldin A (BFA) and nocodazole on *Pth*-GFP transfected cells, which are known to cause microtubule depolymerization and disassembly of the Golgi apparatus, respectively (Supplementary Fig. 5e–j). As a result, we observed that localization of PTH^FVB^-GFP in BFA treated cells was similar to PTH^MSM^-GFP in non-treated cells (Supplementary Fig. 5e–h). Conversely, nocodazole treated *Pth* ^FVB^ and *Pth* ^MSM^ GFP transfected cells exhibited the scattered micro-Golgi distribution of the proteins (Supplementary Fig. 5i,j). Taken together, these results indicate that the majority of PTH^FVB^ is localized in the Golgi and that of PTH^MSM^ is localized in the rER. Finally, to delineate the relationship between the genetic polymorphism in Pro-PTH and the intracellular PTH-GFP levels, we conducted the pulse-chase experiment with cycloheximide (Chx), a well-known inhibitor of protein synthesis to measure protein turnover. As PTH^FVB^ expression was lower than PTH^MSM^ (Fig. 4b), we loaded 2–3-times more protein from PTH^FVB^ transfected cells in the gel compared to PTH^MSM^ to adjust the protein levels between the two alleles. As a result, substitution of Val residue 28 with Met in Pro-PTH (PTH^MSM^) significantly increased the intracellular protein half-life of PTH-GFP (Fig. 4g,h and Supplementary Fig. 9). We next quantified the extracellular secreted PTH-GFP by immunoprecipitation (IP) assay. *Pth*-GFP transfected NIH-3T3 cells were grown in serum free culture medium for 48 hours. After the incubation time, supernatants were concentrated and used for IP. Consequently, secreted PTH-GFP was higher in *Pth* ^MSM^ than in *Pth* ^FVB^ (Fig. 4i and Supplementary Fig. 10). Furthermore, we measured basic-fluorescence of turboGFP in the supernatants of each *Pth* allele-transfected cells. We detected that the *Pth* ^MSM^ allele produced a stronger fluorescent intensity by micro-plate reader when compared with the *Pth* ^FVB^ allele (Fig. 4j). Taken together, these results suggest that the PTH^MSM^ allele is secreted more efficiently than PTH^FVB^, which could lead to an increase in serum iPTH.

### PTH increases [Ca^2+^]i of keratinocytes and inhibits cell-proliferation

**Figure 5:**
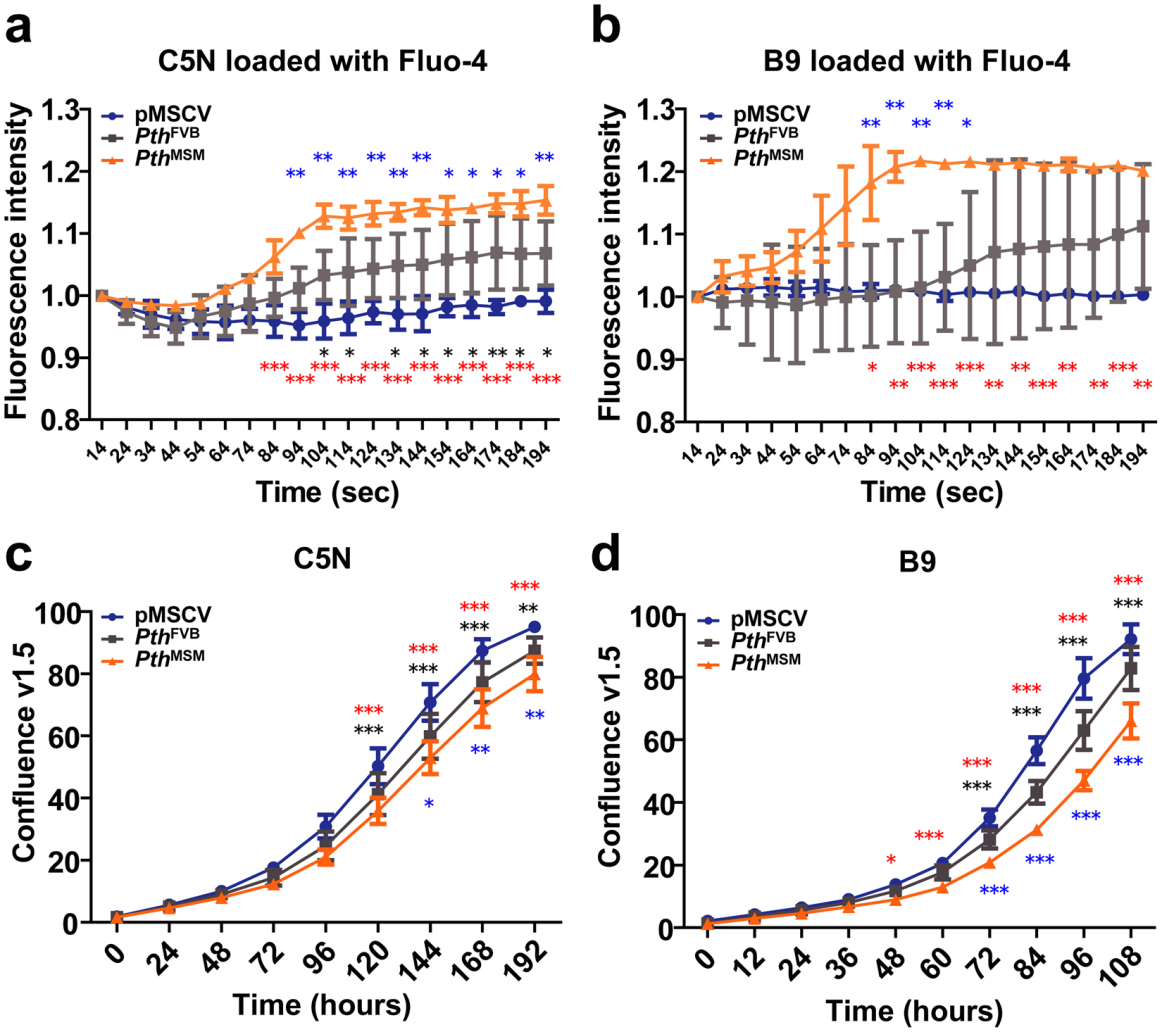
PTH increases intracellular calcium and inhibits cell-proliferation in normal and epidermal tumour cell lines. (**a**,**b**) Intracellular calcium was monitored for 194 seconds after adding serum-free culture medium of pMSCV (control), *Pth* ^FVB^-GFP and *Pth* ^MSM^-GFP to C5N (**a**) and B9 (**b**) cells. C5N is derived from normal mouse keratinocytes. B9 is derived from mouse squamous cell carcinoma cells. The *P-*values were calculated by two-way ANOVA. Black asterisks indicate pMSCV vs *Pth* ^FVB^. Red asterisks indicate pMSCV vs *Pth* ^MSM^. Blue asterisks indicate *Pth* ^FVB^ vs *Pth* ^MSM^ (***P < 0.001, **P < 0.01, *P < 0.05). (**c**,**d**) Cell proliferation assay after adding secreted PTH-GFP from *Pth* ^FVB^- and *Pth* ^MSM^- transfected NIH-3T3 cells. These collected supernatants were mixed with new DMEM (1:1) and added to 1 × 10^3^ cells of C5N (**c**) or B9 (**d**) seeded on 96 well plates. Proliferation curves represent mean confluence values ± SEM of triplicates. The *P-*values were calculated by two-way ANOVA. Black asterisks indicate pMSCV vs *Pth* ^FVB^. Red asterisks indicate pMSCV vs *Pth* ^MSM^. Blue asterisks indicate *Pth* ^FVB^ vs *Pth* ^MSM^ (***P < 0.001, **P < 0.01, *P < 0.05). (**a**–**d**) Error bars are standard deviation (SD). Asterisks indicate significant differences.

Previously, it was reported that the tuberoinfundibular peptide of 39 residues (TIP39) is the third member of the PTH ligand family, which increases intracellular calcium ([Ca^2+^]i) in keratinocytes^33^. To address whether the secreted PTH increases [Ca^2+^]i in keratinocytes, we first monitored [Ca^2+^]i following the addition of hPTH1-34 agonist (1 nM and 1 μM) in the culture medium of C5N (derived from normal mouse keratinocytes) and B9 (derived from mouse squamous cell carcinoma) cells. As a result, hPTH1-34 increased [Ca^2+^]i in a dose-dependent manner under basal growth conditions containing low calcium concentrations in the medium, (Supplementary Fig. 6a,b). We further explored the intracellular calcium change of C5N and B9 cells after adding the supernatants from *Pth* ^FVB^- and *Pth* ^MSM^-transfected cells. Consequently, we observed that *Pth* ^MSM^ enhanced upregulation of [Ca^2+^]i compared with the *Pth* ^FVB^ allele in C5N (Fig. 5a) and B9 (Fig. 5b) cells. Consistent with the effects of PTH on calcium homeostasis in keratinocytes, a change in cell-proliferation was apparent in C5N and B9 cells when exposed to hPTH1-34 for 100~200 hours in a dose-dependent manner (Supplementary Fig. 6c,d). Similarly, we confirmed that supernatants from *Pth*-transfected cells inhibited cell-proliferation in C5N (Fig. 5c) and B9 (Fig. 5d) cells. These results indicate that higher secreted levels of PTH-GFP from the *Pth* ^MSM^ allele promote stronger acceleratory intracellular calcium accumulation and inhibitory effects on cell proliferation.

### Pth^MSM^-Tg mice show enhanced keratinocyte differentiation

**Figure 6:**
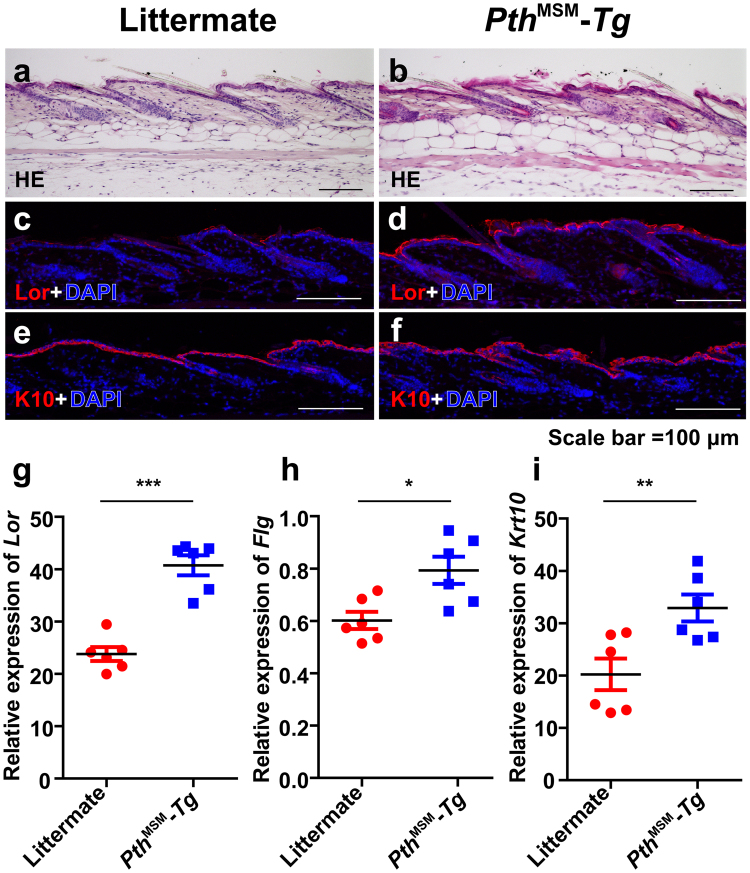
The expressions of cell-differentiation markers are elevated in dorsal back skin of *Pth* ^MSM^-*Tg* mice. (**a**,**b**) Dorsal back skin HE sections of 8-week-old littermates (n = 5) (**a**) and *Pth* ^MSM^-*Tg* mice (n = 5) (**b**) are shown. (**c–f**) Representative immunostaining patterns of anti-Loricrin antibody (Lor) (**c**,**d**) and anti-Kratin10 antibody (Krt10) (**e**,**f**) positive cells (red) in the skin from control littermates (**c**,**e**) and *Pth* ^MSM^-*Tg* mice (**d**,**f**). Cells were counterstained with DAPI (blue). (**g–i**) The mRNA expression levels of *Lor* (**g**), *Flg* (**h**) and *Krt10* (**i**) are shown for each littermate (red) and *Pth* ^MSM^-*Tg* mouse (blue) at the age of 2 months. The *Lor*, *Flg* and *Krt10* transcript levels are shown relative to the transcript levels of *Gapdh*. The *P-*values were calculated by *t*-test (*P < 0.05, **P < 0.01, and ***P < 0.001). (**g**–**i**) Error bars are standard deviation (SD). Asterisks indicate significant differences. Horizontal bold lines indicate mean values. Each dot indicates each experiment. Scale bars, 100 μm.

Finally, we investigated the dorsal back skin of 8-week-old female *Pth* ^MSM^ *-Tg* mice and their littermates (Fig. 6a–f). Consistent with the effects of high [Ca^2+^]i promoting differentiation of keratinocytes^33^, the epidermis of *Pth* ^MSM^ *-Tg* mice was observed to be thicker than that of their littermates (Fig. 6a,b). We then conducted immunofluorescence analysis with Loricrin as a cornified layer marker and Keratin10 as a granular layer marker. As a result, expression of both markers increased in *Pth* ^MSM^ *-Tg* epidermis when compared with littermates. We then quantified mRNA levels of *Lor*, *Flg* (as a terminal differentiation marker) and *Krt10*. As a result, mRNA levels of all three markers were also upregulated in *Pth* ^MSM^ *-Tg* skin (Fig. 6g–i). Immunofluorescence analysis revealed that the number of cell proliferation marker Ki-67-positive cells was also lower in keratinocytes from *Pth* ^MSM^ *-Tg* than in littermate controls at two days after short term TPA treatment (Supplementary Fig. 7). We analyzed papillomas from *Pth* ^MSM^ *-Tg* and sub-congenic mice by HE staining. However, HE staining of these papillomas showed no significant morphological differences (Supplementary Fig. 8). Collectively, these findings suggest that iPTH increases [Ca^2+^]i in keratinocytes and differentiates keratinocytes, resulting in inhibition of cell proliferation and resistance to skin carcinogenesis.

## Discussion

**Figure 7:**
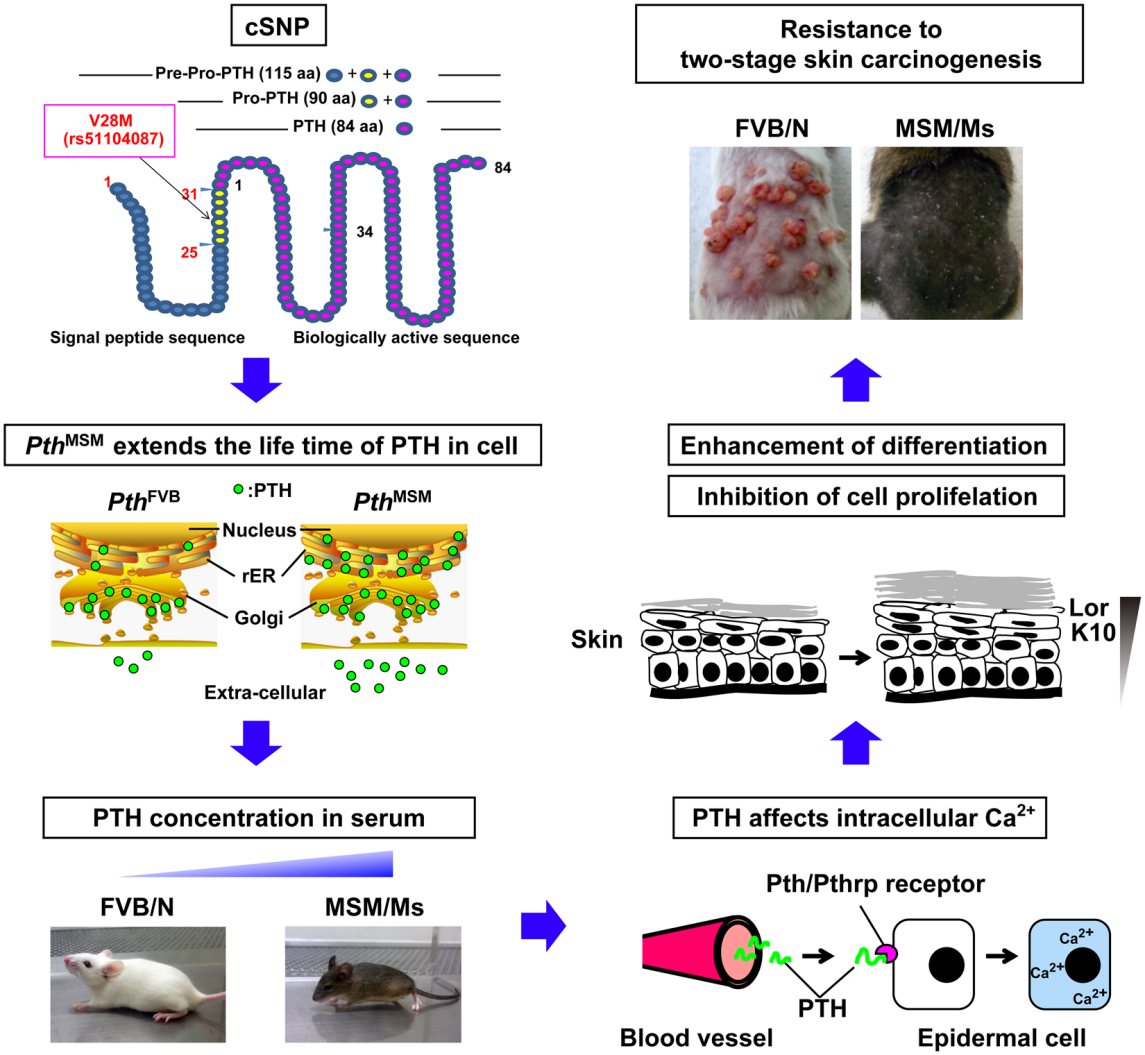
A schematic drawing of the mechanisms of a genetic polymorphism in *Pth* conferring resistance to two-stage skin carcinogenesis in mice. This figure shows a flow chart of how a genetic polymorphism in *Pth* changes the resistance to the chemically induced skin carcinogenesis between FVB/N and MSM/Ms. Secreted PTH is increased by elongating the protein half-life in MSM, which could lead to an increase in serum iPTH. Furthermore, serum intact-PTH increases the intracellular calcium in keratinocytes, which enhances the differentiation and suppresses proliferation of keratinocytes by the endocrine route. As a result, a polymorphism in *Pth* leads to resistance to chemically induced skin carcinogenesis in MSM.

In this study, we have demonstrated that circulating PTH levels modify the development of chemically induced skin papillomas, and account, at least in part, for the effects of *Stmm1*. We measured serum intact-PTH (iPTH) in MSM and FVB strains. Surprisingly, we found significantly higher iPTH levels in sera from MSM mice than in sera from FVB mice. This difference in sera iPTH levels was observed in MSM-BAC transgenic (*Pth* ^MSM^-*Tg*) mice and was associated with resistance to skin carcinogenesis. A genetic variant in PTH between MSM (i.e. methionine) and FVB (i.e. Valine) occurs in the Pro-PTH region (rs51104087, amino acid position 28). *In vitro* studies showed that this difference affected PTH protein stability and the *Pth* ^MSM^ allele increased intracellular calcium concentration compared with *Pth* ^FVB^. Finally, we elucidated that the expression of keratinocyte differentiation markers was increased and the proliferation of cells was decreased in *Pth* ^MSM^-*Tg* mice (Fig. 7). All these results suggest that *Pth* is an important modifier gene of skin tumour development.

We have detected a well-conserved Val/Met polymorphism in the Pro-PTH region of the mouse *Pth* gene between FVB and MSM. The Pro-PTH sequence is conserved in mammals, which is important for efficacy of the signal peptide sequence *in vitro* ^34^ and *in vivo* ^35^. Therefore, we hypothesized that cSNP in Pro-PTH could influence the protein posttranslational modification and increase secreted PTH. Physiologically, nascent PTH is translated as a pre-pro protein. The pre-sequence is a 25 residue N-terminal signal sequence necessary for efficient transport into the endoplasmic reticulum (ER). This signal sequence is rapidly cleaved off co-translationally in the rough ER and the resulting Pro-PTH peptide is transported to the Golgi apparatus^36^ where the pro sequence is cleaved. In this study, despite similar mRNA expression levels, we observed that PTH fusion GFP protein was expressed higher in *Pth* ^MSM^ than in *Pth* ^FVB^. In addition, our chemical treatment experiments with *Pth* ^MSM^ and *Pth* ^FVB^ allele-expressing cell lines indicated that the majority of PTH^FVB^ is localized in the Golgi and that of PTH^MSM^ is localized in the rER. However, secreted PTH-GFP was higher in *Pth* ^MSM^ than in *Pth* ^FVB^. This is probably dependent on how long the two PTH forms remain in the rER and Golgi. The main reservoir of PTH^MSM^ is obviously the rER. It is expected that once PTH^MSM^ is transported to the Golgi, it is secreted shortly thereafter. On the other hand, the main reservoir of PTH^FVB^ is the Golgi. It seems likely that PTH^FVB^ stays in the Golgi longer than PTH^MSM^. This may be the reason for the differences in the localization and in the secretion levels between the two alleles. Nevertheless, our findings indicate that the *Pro-pth* ^MSM^ polymorphism can alter the transport and secretory efficacy of the PTH peptide.

PTH is a classical endocrine hormone that was first identified more than 80 years ago as a key regulator of blood calcium levels^37^. Serum PTH is a sensitive indicator of calcium and vitamin D deficiency. Vitamin D and calcium are known to regulate differentiation and proliferation of keratinocytes; they may potentially have roles in suppressing carcinogenesis in squamous epithelium^38^. Indeed, the importance of the vitamin D receptor (VDR) in regulating cellular proliferation and differentiation was verified when the skin of mice lacking the VDR was reported to be susceptible to tumour formation^38^. In addition, knockout of the calcium sensing receptor in addition to VDR accelerated the development of skin tumours^39^. However, we did not detect differences in either serum calcium or 1,25(OH)_2_D in MSM, *Pth* ^MSM^-*Tg* and *Pth* ^+/−^ KO mice (Figs 1, 2 and 3). These results indicate PTH confers resistance to skin tumours independently from serum calcium and vitamin D.

Genes related to the PTH family influence skin carcinogenesis and development, but the full function of the high-serum PTH in cutaneous biology remains incompletely understood. Previously studies have reported that the PTH signaling led to modulation of epidermal cells and hair growth in rodents^27^. Furthermore, PTH agonist (PTH1-34) administration caused a decrease in the number of epidermal cells in mice^23^. There is a significant body of evidence suggesting the potential role of parathyroid hormone receptor ligands. We checked *Pth* expression patterns *in vivo*. *Pth* was not expressed in the skin and specific localization of *Pth* mRNA was observed in the parathyroid tissue (Fig. 1c and Supplementary Fig. 1a). On the other hand, *Pthrp* has been reported to be expressed in the skin^26^. In addition, PTH as well as PTHrP interacts with PTH1R in target tissues binding with equal affinity^28^. Previously, it was reported that *Pthrp* is a modifier gene of skin carcinogenesis in the Car mice line^40, 41^. However, we did not detect differences in *Pthrp* and *Pth1r* mRNA expression between FVB and MSM mice. Taken together, these results indicate that the high-serum iPTH has a modulating function of skin carcinogenesis independently from PTHRP by directly binding to its receptor in keratinocytes. We demonstrated that high serum PTH in *Pth* ^MSM^-*Tg* decreased skin papillomas. Contrarily, *Pth* ^+/−^ KO mice exhibited increased papillomas when subjected to the DMBA-TPA skin carcinogenesis experiments. The differences in sera iPTH levels we observed in *Pth* ^MSM^-*Tg* and *Pth* ^+/−^ KO were associated with their resistance and susceptibility to skin carcinogenesis. These results indicate that high-serum PTH functions as a tumour suppressor during early papillomagenesis. However, the PTH type 2 receptor (PTH2R), which can be activated by PTH and TIP39 but not by PTHrP, is also present in keratinocytes^33, 42^. Sato *et al*. (2016) reported that TIP39 and its receptor in the epidermis influence keratinocyte differentiation by increasing intracellular calcium. Similarly, we report that PTH increases intracellular calcium and differentiates keratinocytes *in vitro* and *in vivo*. Furthermore, PTH treatment induces G0/G1 arrest of epithelial intestinal cells and changes the expression of proteins involved in cell cycle regulation via the PKC signaling pathway^43^. These results indicate that serum PTH exerts anti-proliferative and pro-differentiating effects on keratinocytes and inhibits papilloma formation in mouse skin subjected to the topical two-stage carcinogenesis protocol.

In summary, we have shown that *Pth* accounts, at least in part, for the effects attributed to *Stmm1b*. Our data demonstrating the association between high serum PTH levels and skin tumour suppression in a two-stage skin carcinogenesis model underscore the potential therapeutic impact of using PTH or PTH analogues in the prevention and treatment of skin cancer.

## Methods

### Mouse strains

The control C57BL/6 J and FVB/N mice were purchased from CLEA Japan (Tokyo, Japan). To produce genetic crosses, the MSM/Ms^44^ were obtained from the National Institute of Genetics and maintained at the Chiba Cancer Center Institute. In a previous large F_1_ backcross study using [(FVB/N × MSM/Ms) × FVB/N], papilloma resistance loci were identified by QTL analysis^17, 45^. Resistant F_1_ backcross mice were selected for further backcrossing to FVB/N mice over at least 10 generations, ultimately leading to *Stmm1* congenic mice^18^ to perform the congenic mapping. In addition, B6J-*Pth* ^−/−^ mice^19^ were obtained from a co-author Dr. Andrew C. Karaplis, which were maintained at the Chiba Cancer Center Institute by mating homozygous male mice with heterozygous female mice.

### Skin carcinogenesis

7,12-Dimethylbenz(a)anthracene (DMBA) was purchased from Sigma Japan, and 12-*O*-tetradecanoylphorbol-13-acetate (TPA) was purchased from Calbiochem-Merck Millipore (Darmstadt, Germany). DMBA is used as a carcinogen and TPA as a promoter. The congenic and *Tg* mice were treated according to the two-stage carcinogenesis protocol. At 8–10 weeks of age, the backs of mice were carefully shaved with an electric clipper. Two days after shaving, a single dose of DMBA (25 μg per mouse in 200 μl of acetone) was applied to shaved dorsal back skin. One week after initiation, tumours were promoted with TPA (10 μg per mouse in 200 μl of acetone) twice weekly for 20 weeks. KO mice were treated according to the modified two stage carcinogenesis protocol^46^. Two days after shaving, DMBA (25 μg per mouse in 200 ul of acetone) was applied to shaved dorsal back skin. Three days after the first DMBA treatment, TPA (10 μg per mouse in 200 μl of acetone) was applied. After four rounds of this single DMBA and TPA treatment, mice were treated with TPA twice weekly for 20 weeks. Papilloma number and size (mm in diameter) of each papilloma was recorded from 8 weeks up to 20 weeks.

### Serum biochemistry

Blood samples were collected by cardiopuncture for serum PTH, calcium and 1,25(OH)_2_ vitamin D_3_ measurements. PTH was measured using a mouse parathyroid hormone ELISA kit (Cusabio Biotech Co., Ltd., Wuhan, China). Total calcium was measured using Metallo assay Calcium LS (oCPC) kit (Metallogenics Co., Ltd., Chiba, Japan). The serum levels of 1,25(OH)_2_D and 25(OH)D were measured by RIA (SRL Inc., Tokyo, Japan).

### Generation of MSM-BAC transgenic mice

The MSM BAC clones (MSMg01-047A16 and MSMg01-466J23) were chosen by the NIG Mouse Genome Database Map view tool (http://molossinus.lab.nig.ac.jp/msmdb/index.jsp). The BAC clones were purchased from RIKEN bio resource center DNA BANK (http://dna.brc.riken.jp/en/MSMBACen.html), and the bacteria were grown and BAC DNA isolated according to the standard protocol of QIAGEN Plasmid Maxi Kit. DNA purification and insert check were performed by PCR amplification (Arntl-F and R, Btbd10-F and R, Pth-F3 and R1, 047AT7-F and R, 047ATJ-F and R, 466JT7-F and R, 466JTJ-F and R; Table S1), with an additional step of phenol-chloroform-isoamylalcohol precipitation to separate DNA from precipitated proteins. DNA was ethanol precipitated and diluted in TE buffer (10 mM Tris-HCl, 0.1 mM EDTA, pH 8.0). DNA was injected into pronuclei of fertilized oocytes of FVB/N mice, which were transferred into pseudopregnant female mice. Genomic DNA was extracted using PCI standard protocol from the tail of the offspring and genotyping was performed. The BAC DNA region was amplified by using a Prime Taq (GeNet Bio., Daejeon, Korea) and a primer pair (Table S1). After being bred with wild-type (WT) FVB males, positive MSM-*Pth* second-generation offspring were crossed among themselves to receive a higher ratio of MSM-*Pth*-positive mice. Genomic DNA of transgenic mice was isolated from tail samples using a standard phenol-chloroform extraction followed by 70% alcohol precipitation. Genotyping for the *Pth* variant (-82A/G; rs51104087) was carried out using Custom TaqMan SNP Genotyping Assays (Applied Biosystems, Foster City, CA, USA). The primer sequences were 5′-CTGTCTTCTTACCCAAAC-3′ (forward) and 5′-GTGCATAAGCTGTATTTCA-3′ (reverse). The TaqMan minor groove binder probe sequences were 5′-CTCTTCCTCATGGGTTTCCCA-3′ and 5′-CTCTTCCTCACGGGTTTCCCA-3′. The probes were labeled with the TaqMan fluorescent dyes VIC and FAM, respectively. The PCR was conducted in a total volume of 15 *μ*L using the following amplification protocol: denaturation at 95 °C for 30 sec, followed by 40 cycles of denaturation at 94 °C for 5 sec, followed by annealing and extension at 56 °C for 40 sec. All animal experiments were approved by Chiba Cancer Center and Animal Care and Tokyo Metropolitan Institute of Medical Science Use Committee.

### Mutagenesis and construction of expression plasmids

The mouse *PTH* cDNA vector pCMV6-AC-GFP was purchased from OriGENE (Rockville, MD, USA). This sequence was designed from *Pth* transcript sequence (Ensemble, ENSMUST00000079793). To introduce a mutation of V28M in the *Pth* gene, template pCMV6-AC-GFP was prepared by the PrimeSTAR mutagenesis basal kit (Takara Bio Inc., Kusatsu, Japan) according to the instructions provided by the manufacturer. The following primers were designed to introduce the mutations of V28M into the *Pth* gene: for PTH-MutaF: 5′-GAAACCCATGAGGAAGAGAGCTGTCAG-3′ (sense) and PTH-MutaR: 5′-TTCCTCATGGGTTTCCCATCCGTTTGG-3′ (anti-sense). PCR amplification was performed using PrimeSTAR Max DNA Polymerase (Takara Bio Inc.) and consisted of 35 cycles at 98 °C for 10 sec, 55 °C for 15 sec, and 72 °C for 40 sec. To permit puromycin selection of stable transfectants, a turboGFP fragment containing the entire *Pth* coding sequence was subcloned into the retroviral mammalian expression vector pMSCVpuro. The following primer sets were designed to subclone into pMSCVPuro: for PTH_bgl2_F: 5′-TTTAGATCTATGATGTCTGCAAACACCG-3′ (sense) and Puro_Eco_R: 5′-TTTGAATTCATTAGGACAAGGCTGGTGGG-3′ (antisense). The *Pth* V28M mutation was confirmed by DNA sequencing of the products amplified by a following primer sets: for PMSCV-F2: 5′-CCTACATCGTGACCTGGGAAG-3′ (sense), F3-pth: 5′-GCA AAC ACC GTG GCT AAA GT-3′ (sense), F2-pth: 5′-CAT GGA GAG GAT GCA ATG G-3′ (sense) and PMSCV-R: 5′-GAGACGTGCTACTTCCATTTGTC-3′ (anti-sense).

### Cell culture and transfection

NIH-3T3, PlatE, C5N and B9 cells were grown in Dulbecco’s modified Eagle’s medium (DMEM) supplemented with 10% fetal bovine serum and 1% penicillin/streptomycin at 37 °C in a humidified atmosphere of 95% air and 5% CO_2_. Retrovirus expressing *Pth* was constructed using a pMSCV retroviral expression system (Takara Bio Inc.). Pth^FVB^-turboGFP and Pth^MSM^-turboGFP were cloned into pMSCVpuro, respectively. The PlatE cells were transfected with pMSCV-Pth^FVB^-turboGFP or pMSCV-Pth^MSM^-turboGFP by FuGENE6 transfection reagent (Promega Co, Madison, WI, USA) following the manufacturer’s instructions when the cells were 80% confluent. Vector empty control PlatE cells were also transfected with pMSCVpuro by FuGENE6. After 48 hours, the supernatants were collected. NIH-3T3 (1 × 10^6^/10 cm dish) cells were incubated with these supernatants in the presence of 10 µg/ml polybrene (Merck Millipore) for 24 hours. The transfection efficiency was approximately 100% as assessed by fluorescence microscopy for turboGFP, and by immunostaining and quantitative real-time PCR of *Pth* after treatment with puromycin (3 µg/ml) (Sigma-Merck Millipore) for 2–3 weeks.

### Quantitative real-time RT-PCR

Total RNA from cells and tissues (whole P0 pups and dorsal back skins of 3-month-old mice) were extracted using the RNAeasy kit (QIAGEN, Hilden, Germany) following the manufacturer’s protocol. cDNAs were synthesized using iScript Select cDNA Synthesis Kit (Bio-Rad, Hercules, CA, USA). Quantitative PCR was performed with *Pth* ^47^, *Pthrp*, *Pthr1*, *Lor* ^48^, *Flg* ^49^ and *Krt10* ^50^-specific primers (Table S2) and SsoFast EvaGreen Supermix With Low ROX (Bio-Rad) on Applied Biosystems 7500 (Thermo Fisher Scientific Inc., Waltham, MA, USA). *Actb* and *Gapdh* were used as internal controls (Table S2). Relative expression was calculated based on the threshold cycle with different efficiency of each primer.

### *In situ* hybridization

Paraffin embedded blocks and sections of E18.5 and mouse skin for ISH were obtained from Genostaff Co., Ltd. The E18.5 sections and mouse skin were dissected, fixed with Tissue Fixative (Genostaff), embedded in paraffin by their proprietary procedures, and sectioned at 4-µm. For ISH, tissue sections were de-waxed with xylene, and rehydrated through an ethanol series and PBS. The sections were fixed with 4% para-formaldehyde in PBS for 15 min and then washed with PBS. The sections were treated with 10 µg/ml ProteinaseK in PBS for 30 min at 37 °C, washed with PBS, re-fixed with 4% para-formaldehyde in PBS, again washed with PBS, and placed in 0.2 N HCl for 10 min. After washing with PBS, the sections were acetylated by incubation in 0.1 M tri-ethanolamine-HCl, pH 8.0, 0.25% acetic anhydride for 10 min. After washing with PBS, the sections were dehydrated through a series of ethanol. Hybridization was performed with probes at concentrations of 300 ng/ml in the Probe Diluent (Genostaff) at 60 °C for 16 hrs. After hybridization, the sections were washed in 5 × HybriWash (Genostaff), equal to 5xSSC, at 60 °C for 20 min and then in 50% formamide, 2 × HybriWash at 60 °C for 20 min, followed by RNase treatment in 50 µg/ml RNaseA in 10 mM Tris-HCl, pH8.0, 1 M NaCl and 1 mM EDTA for 30 min at 37 °C. Then the sections were washed twice with 2 × HybriWash at 60 °C for 20 min, twice with 0.2 × HybriWash at 60 °C for 20 min, and once with TBST (0.1% Tween20 in TBS). After treatment with 1xG-Block (Genostaff) for 15 min at RT, the sections were incubated with anti-DIG AP conjugate (Roche) diluted 1:2000 with × 50 G-Block (Genostaff) in TBST for 1 hr at RT. The sections were washed twice with TBST and then incubated in 100 mM NaCl, 50 mM MgCl_2_, 0.1% Tween20, 100 mM Tris-HCl, pH 9.5. Staining reactions were performed with NBT/BCIP solution (Sigma-Merck Millipore) overnight and then washed with PBS. The sections were counterstained with Kernechtrot stain solution (Mutoh, Tokyo, Japan), and mounted with CC/Mount (DBS).

### Protein extraction and immunoblotting

The proteins were extracted from different cells using T-PER Protein Extraction Reagent (Thermo Fisher Scientific Inc.). Protein concentrations were quantified with Quick Start Bradford Protein Assay (Bio-Rad). Denatured proteins (20 µg) were then analyzed using 10% e-PAGELs (ATTO Corporation, Tokyo, Japan). After electrophoresis, proteins were transferred to a polyvinylidene difluoride (PVDF) membrane (Merck Millipore). The membrane was blocked with 0.5% skim milk in phosphate buffered saline solution (pH 7.6) containing 0.1% Tween-20 (PBS/T), and then SNAP i.d. 2.0 Protein Detection system (Merck Millipore). The membrane was subsequently stripped and blotted with polyclonal rabbit anti-turboGFP antibody (Evrogen JSC, Moscow, Russia) (1:2,000 dilutions) and polyclonal rabbit anti-ACTIN (Sigma-Merck Millipore) (1:2,000 dilutions) to control for protein loading. HRP-conjugated secondary antibodies (Cell Signaling, Tokyo, Japan) were used at a dilution of 1:2,000 and developed using the ECL Prime Western Blotting Detection Kit (GE Healthcare, Buckinghamshire, UK). Exposure for chemiluminescent samples or membrane analysis for the blots was performed by LAS4000 (GE Healthcare).

### Immunoprecipitation

IP procedures were performed at 4 °C unless otherwise indicated, using a Pierce spin column which can be capped and plugged with a bottom plug for incubation or unplugged to remove the supernatant by centrifugation at 1000 g for 1 min. The binding of turboGFP antibody to protein A/G agarose was performed with the protocol described in Pierce crosslink immunoprecipitation kits (Thermo Fisher Scientific Inc.). Protein A/G agarose slurry (20 µl) was washed twice with 200 µl PBS buffer, and incubated with 100 µl turboGFP antibody prepared in PBS (10 µl turboGFP antibody (1 µg/µl) + 85 µl H_2_O + 5 µl 20 × PBS) at room temperature for 60 min on a mixer. In parallel, 100 µl of anti-rabbit IgG peroxidase secondary antibody (1 µg/µl) with the same concentration of IgG was similarly prepared as the negative control. The supernatant was discarded and the beads were washed three times with 300 µl PBS, followed by incubation with 50 µl of DSS solution (2.5 µl 20 × PBS + 38.5 µl H_2_O + 2.5 mM DSS in DMSO) at RT for 60 min on a mixer. After removing the supernatant, the beads were washed three times with 50 µl of 100 mM glycine (pH 2.8), twice with 300 µl of PBS buffer containing 1% NP-40, then once with 300 µl of PBS. The antibody-cross-linked beads were incubated overnight at 4 °C with 800 µg of protein from serum-free culture supernatant of NIH3T3-pMSCV, -pMSCV-Pth^FVB^-turboGFP and -pMSCV-Pth^MSM^-turboGFP, which were pre-cleared with control agarose resin (Pierce) at 4 °C for 60 min on a shaker. After the incubation, the beads were washed three times with lysis/wash buffer and the eluted complex was subjected to SDS-PAGE separation for Immunoblotting.

### Immunofluorescence

The dorsal back skin or cells were fixed in 4% paraformaldehyde at 4 °C overnight or at room temperature for 30 min. Dehydrated samples were embedded in paraffin and sectioned to 10-µm slices. The endogenous peroxidase activities in the specimens were blocked by treatment with 0.3% H_2_O_2_ and then were rinsed with PBS. Sections were incubated with primary antibodies diluted in blocking buffer overnight at 4 °C. The following primary antibodies were used: rabbit anti-turboGFP (1:200 dilution, Evrogen JSC), rabbit anti-keratin 14 (1:500 dilution, Covance Inc., Princeton, NJ, USA), rat anti-Ki-67 (1:200 dilution, DakoCytomation-Agilent Technologies, Santa Clara, CA, USA), rat anti-Grp94 (1:100, Abcam, Cambridge, UK), rabbit anti-keratin 10 (1:500 dilution, Covance Inc), rabbit anti-loricrin (1:200 dilution, Covance Inc.) Secondary antibodies were Alexa Fluor 488-conjugated anti-rat antibody (1:100, Molecular Probes, Invitrogen-Thermo Fisher Scientific Inc.) and Alexa Fluor 568-conjugated anti-rabbit antibody (1:100, Molecular Probes, Invitrogen-Thermo Fisher Scientific Inc.). Nuclei were counterstained with Hard Set Mounting Medium with DAPI (Vector Laboratories, Burlingame, CA, USA). All fluorescence images were obtained with a Leica TCS SPE confocal microscope equipped with a DMI4000B (10 ×/0.40, 20 ×/0.70, and 40 ×/1.25 oil immersion objective).

### Chx chase assay and co-culture with several inhibitors

To initiate the chase, cycloheximide was added to a final concentration of 0.15 mg/ml. After cycloheximide addition, NIH3T3-pMSCV, -pMSCV-Pth^FVB^-turboGFP and -pMSCV-Pth^MSM^-turboGFP cells were immediately harvested. Cells were harvested in this way for each subsequent time point. Cells were collected by centrifugation and washed once in PBS, and lysates were prepared as described^12^. The amounts of PTH-GFP at a given chase time were expressed relative to that observed at time zero. Replicates of multiple experiments were averaged and the error is represented as the standard deviation. Error bars are included for all data points. For experiments with an inhibitor of the microtubule transport, cultures of NIH3T3-pMSCV, -pMSCV-Pth^FVB^-turboGFP and -pMSCV-Pth^MSM^-turboGFP were treated with 5 μg/ml of BFA and Nocodazole dissolved in DMSO at 30 °C for 30 min prior to the cycloheximide-chase assay.

### Measurement of cytoplasmic free Ca^2+^

[Ca^2+^]i was measured by a Calcium Kit Fluo-4 (Dojindo laboratories, Kumamoto, Japan). Before starting the analysis, NIH3T3-pMSCV, -pMSCV-Pth^FVB^-turboGFP and -pMSCV-Pth^MSM^-turboGFP (1 × 10^6^/10 cm dish) cells were cultured in Fibroblast Growth Medium (Promocell) for 48 hours. These collected supernatants were mixed in recording medium. To study the calcium ion influx, cells were incubated with dye loading solution (loading medium with Fluo-4 AM and 1.5 mmol/l Probenecid) at 37 °C for 60 minutes. hPTH1-34 peptide (1 nM and 1 µM) and supernatants were added as indicated in recording medium, and then [Ca^2+^]i was monitored at an emission of 516 nm with excitation of 494 nm using ARVO × 3 (Perkin-Elmer, Massachusetts, MA, USA). Fluo-4 signal intensity were defined as (F-F0)/F0. F was the target fluorescence signal intensity and F0 was the base line calculated by averaging five time points just before the application of the stimulus.

### Cell proliferation assay

Before starting the analysis, NIH3T3-pMSCV, -pMSCV-Pth^FVB^-turboGFP and -pMSCV-Pth^MSM^-turboGFP (1 × 10^6^/10 cm dish) cells were cultured in DMEM for 48 hours. These collected supernatants were mixed with new DMEM (1:1) and it was added to 1000 cells of C5N or B9 seeded on 96 well plates. These plates were placed in an IncuCyteHDTM (Essen Instruments Inc. Michigan, USA) automated incubator microscope. Images were taken every 12 hours (3 images per well, respectively), and cell confluence was calculated per well using the IncuCyte software Confluence v1.5. Proliferation curves represent mean confluence values ± SEM of triplicates.

### Statistical analyses

All results are presented as the mean ± standard deviation (SD). Differences among multiple groups were analyzed by two-way ANOVA. The two groups were compared using the Student’s *t*-test. GraphPad Prism (GraphPad, San Diego, CA, USA) was used to calculate column statistics and compute *P*-values.

### Data availability

The datasets generated during and/or analyzed during the current study are available from the corresponding author upon reasonable request.

### Ethics statement

All procedures involving animals met the guidelines described in the Proper Conduct of Animal Experiments as defined by the Science Council of Japan and were approved by the Animal Care and Use Committee on the Ethics of the Chiba Cancer Center Institute (Permit Number: 16−15). All efforts were made to minimize suffering.

## Electronic supplementary material

Supplementary information
