# Supplementary material for: The parathyroid hormone regulates skin tumour susceptibility in mice
Source: Sci Rep. 2017 Sep 11;7:11208. doi: 10.1038/s41598-017-11561-x (PMC5593851; doi:10.1038/s41598-017-11561-x)
Supplement: Supplementary file 1 — Supplementary information [file 41598_2017_11561_MOESM1_ESM.pdf]

Supplementary information

The parathyroid hormone regulates skin tumour susceptibility in mice

Kazuhiro Okumura[1], Megumi Saito[1], Yasuhiro Yoshizawa[1], Haruka Munakata[1], Eriko Isogai[1], Ikuo Miura[2], Shigeharu Wakana[2], Midori Yamaguchi[3], Hiroshi Shitara[3], Choji Taya[3], Andrew C. Karaplis[4], Ryo Kominami[5], Yuichi Wakabayashi[1]

[1]Department of Carcinogenesis Research, Division of Experimental Animal Research, Chiba Cancer Center Research Institute, 666-2 Nitonacho Chuouku, Chiba 260-8717 Japan.

[2]Technology and Development Team for Mouse Phenotype Analysis: Japan Mouse Clinic, Riken Bioresource Center, 3-1-1 Koyadai, Tsukuba, Ibaraki 305-0074, Japan.

[3]Laboratory for Transgenic Technology, Tokyo Metropolitan Institute of Medical Science, 1-6, Kamikitazawa 2-chome, Setagaya-ku, Tokyo, 156-8506, Japan.

[4]McGill University and Jewish General Hospital, 3755 Côte-Ste-Catherine Road,
E-104
Montreal, Quebec H3T 1E2, Canada.

[5]Department of Molecular Physiology, Niigata University School of Medicine, Asahimachi 1-757, Niigata 951-8510, Japan.

* Corresponding author

E-mail: yuichi_wakabayashi@chiba-cc.jp

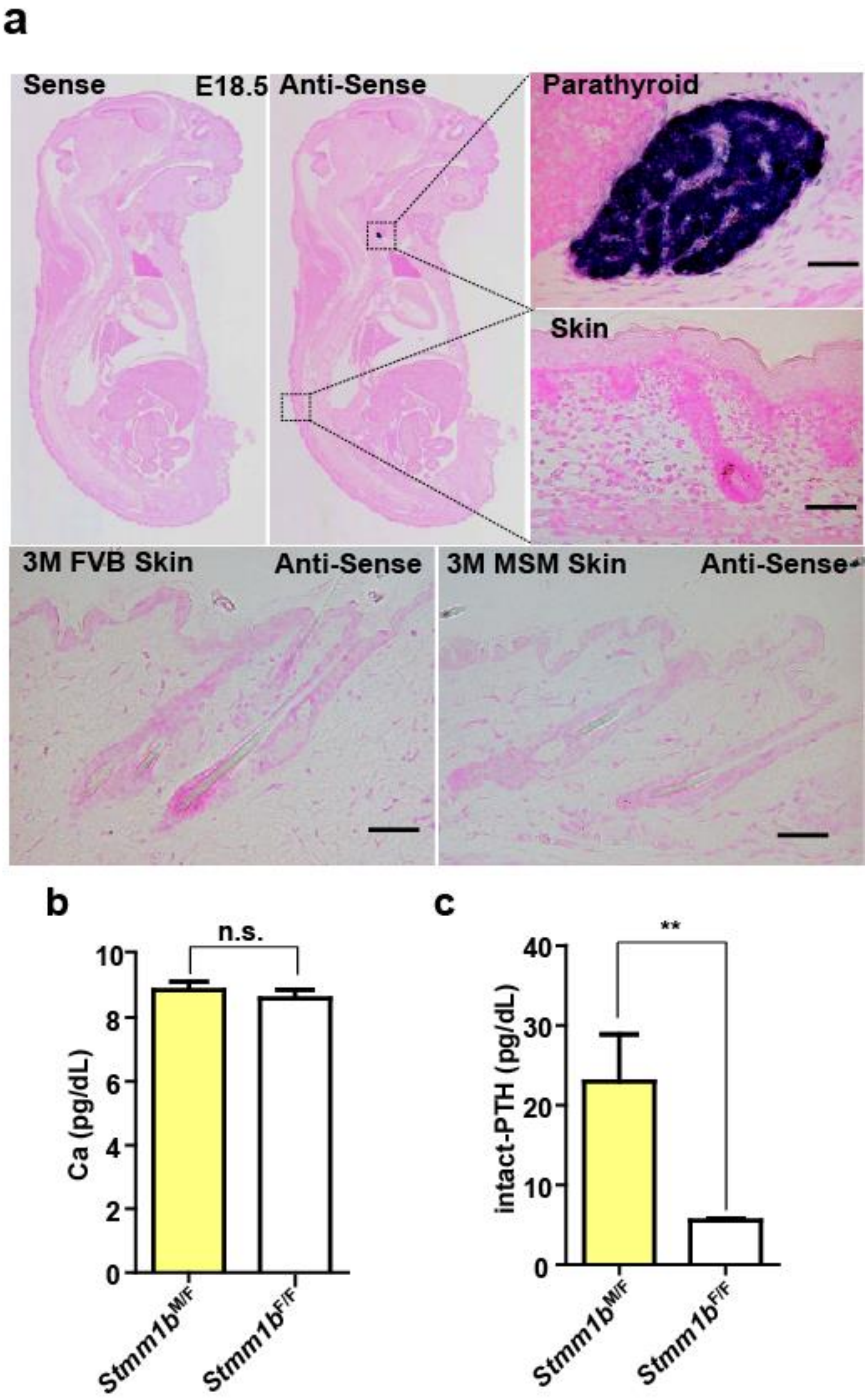
a
Sense
E18.5
Anti-Sense
Parathyroid
Skin
3M FVB Skin
Anti-Sense
3M MSM Skin
Anti-Sense
b
n.s.
Ca (pg/dL)
Stmm1b^M/F
Stmm1b^F/F
c
**
intact-PTH (pg/dL)
Stmm1b^M/F
Stmm1b^F/F

Supplementary Figure S1. *Pth* mRNA localization and *Stmm1b* sub-congenic line shows increase in iPTH in sera. a) Upper panels show *Pth* mRNA expression pattern in E18.5 C57B6/J mice by *in situ* hybridization. Bottom panels indicate *Pth* is not expressed in the dorsal back skins of 3-month-old FVB and MSM mice. Scale bars, 50 µm. **b)** The means of serum calcium concentrations in *Stmm1b*$^{F/F}$ and *Stmm1b*$^{M/F}$ by o-Cresolphthalein-complexone (oCPC method) (n=4 each; female, 3 M). n.s., no significant differences. **c)** The means of serum intact-PTH concentration in *Stmm1b*$^{F/F}$ and *Stmm1b*$^{M/F}$, respectively (n=4 each; female, 3 M) by mouse intact-PTH ELISA. The *P*-value was calculated by *t*-test (**$P < 0.01$). Asterisks indicate significant difference. **b), c)** Error bars are standard deviation (SD). Scale bars, 50 µm.

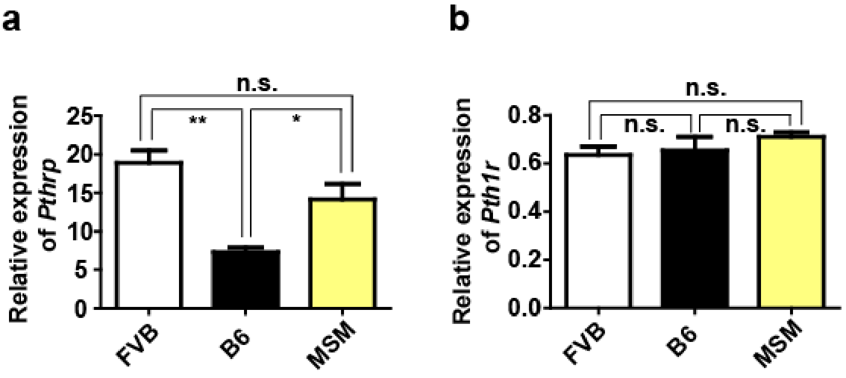

Supplementary Figure S2. The comparison of *Pthrp* and *Pth/Pthrp* receptor mRNA levels among FVB, B6 and MSM strains. a) Comparison of *Pthrp* mRNA in the dorsal back skins among 3-month-old FVB, B6 and MSM mice by qRT-PCR. **b)** Comparison of *Pthr1* mRNA in the dorsal back skins among 3-month-old FVB, B6 and MSM mice by qRT-PCR. The *Pthrp* and *Pth1r* transcript levels are shown relative to the transcript levels of *ActB*. Error bars are the standard deviations (S.D.). The *P*-value was calculated by *t*-test (*$P < 0.05$, **$P < 0.01$). Asterisks indicate significant differences. n.s., no significant differences.

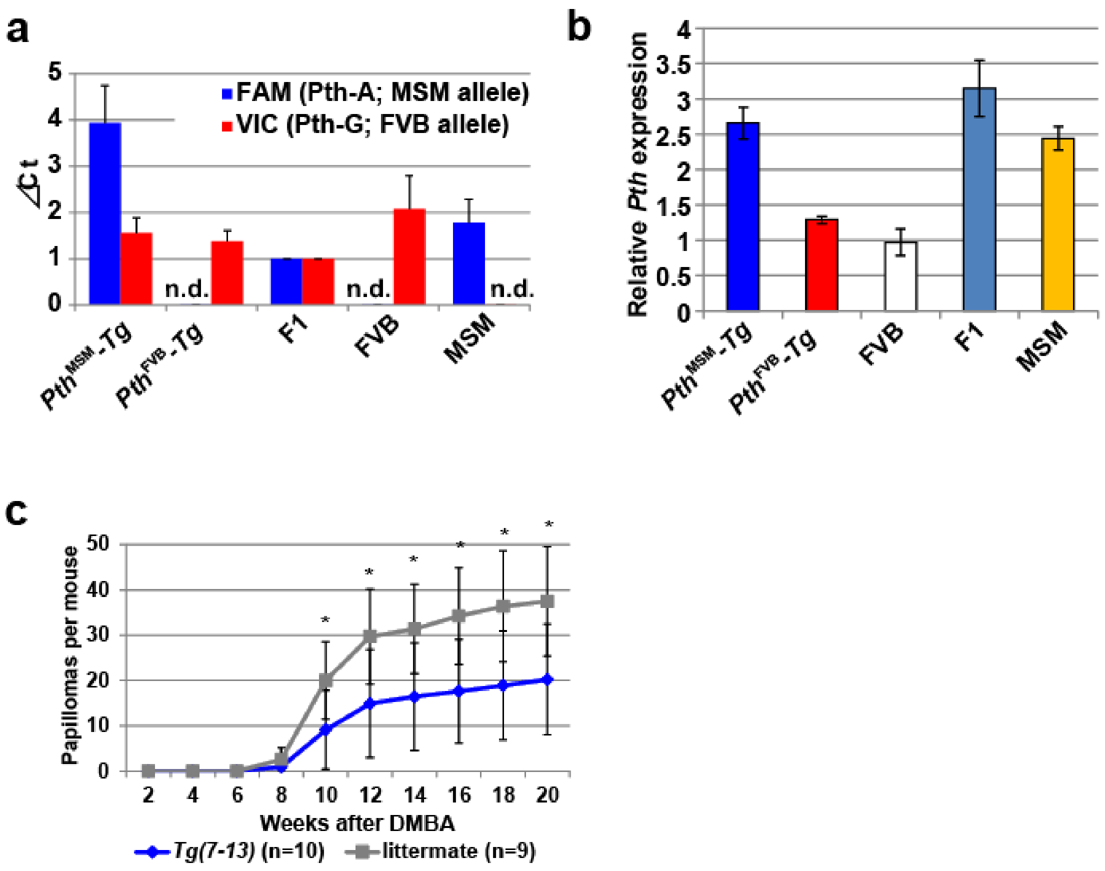

Supplementary Figure S3. The copy number analysis of the *Pth* allele in BAC-transgenic mice and another *Tg* line reveals resistance to skin carcinogenesis. a) Allelic discrimination analysis by TaqMan specific PCR to detect *Pth*^{FVB} and *Pth*^{MSM} alleles. VIC (*Pth*-G) and FAM (*Pth*-A) fluorescence are shown for each probe. F_1 hybrid (FVB×MSM) is a control as one copy for each allele. n.d., not detected. **b)** Comparison of *Pth* mRNA expression levels among *Pth*^{MSM}-*Tg* mice, littermates, FVB/N, F_1 hybrid (FVB×MSM) and MSM/Ms by qPCR. mRNAs were isolated from whole body new born pups (postnatal day 0). **c)** Comparison of the average papilloma number per mouse between *Tg(7-13)* (another line of *Pth*^{MSM}-*Tg*) and littermate mice. The *P*-value was calculated for papilloma number at 10-20 weeks by *t*-test (*$P < 0.01$). Asterisks indicate significant differences. **a)**, **b)**, **c)** Error bars are standard deviation (SD).

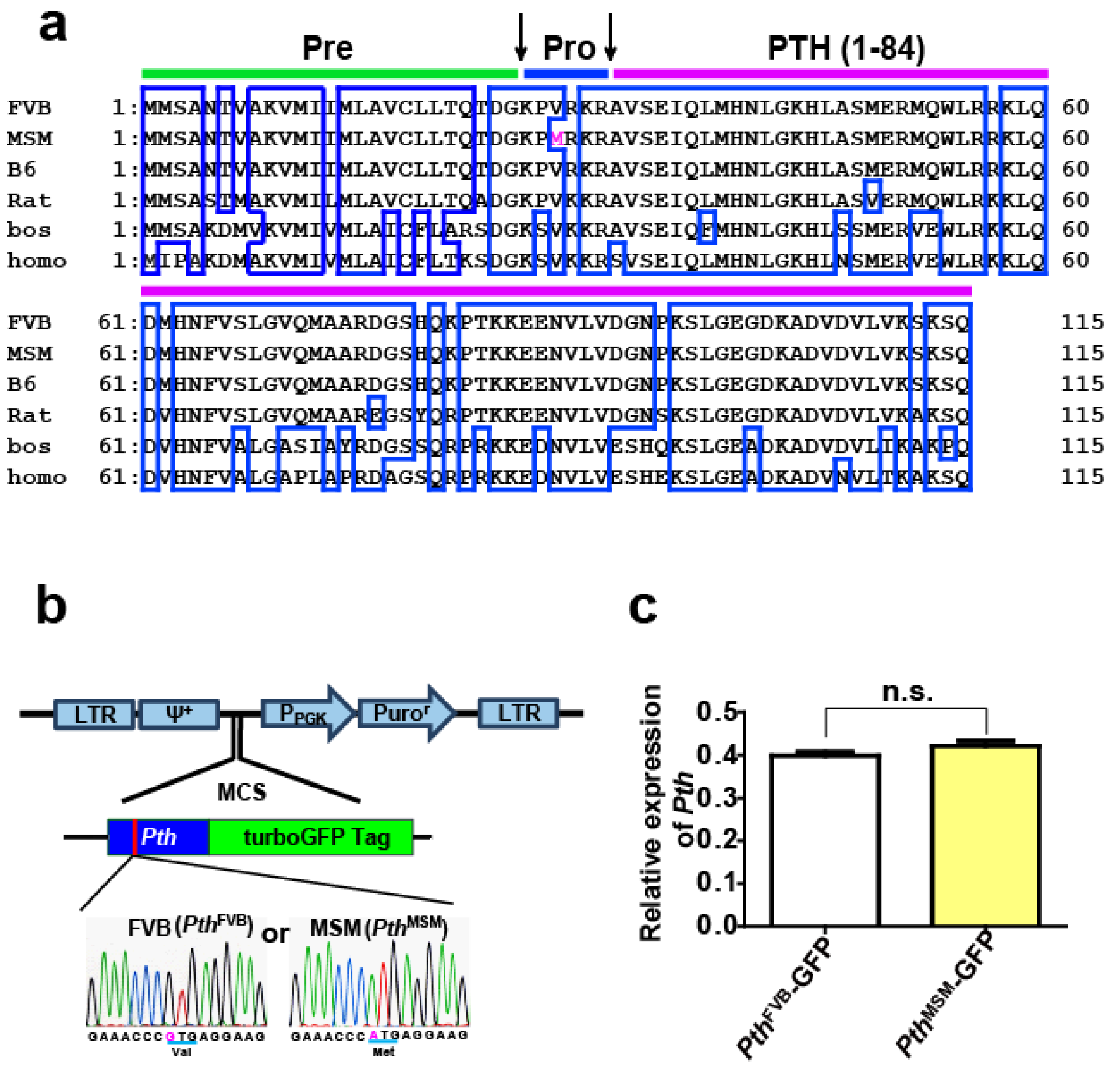

Supplementary Figure S4. Alignment of PTH amino acids of several mammals and a schematic drawing of *Pth*^{FVB} and *Pth*^{Met} constructs. a) Alignment of Pre-Pro-PTH of three mouse strains, rat, caw and human. Green lines indicate the signal peptide sequence region (Pre). A horizontal bold blue line indicates the Pro region. Pink lines indicate the biological active sequence (PTH1-84). Inside the blue lines indicates conserved amino acid sequences. **b)** A schematic drawing of *Pth*-turboGFP Tag constructs for each allele. **c)** qRT-PCR analysis verifying the equivalent transcript levels of *Pth* in *Pth*^{Val} (*Pth*^{FVB} allele)- and *Pth*^{Met} (*Pth*^{MSM} allele)-transfected NIH-3T3 cells. The *P*-value was calculated by *t*-test. n.s., no significant differences. Error bars are standard deviation (SD).

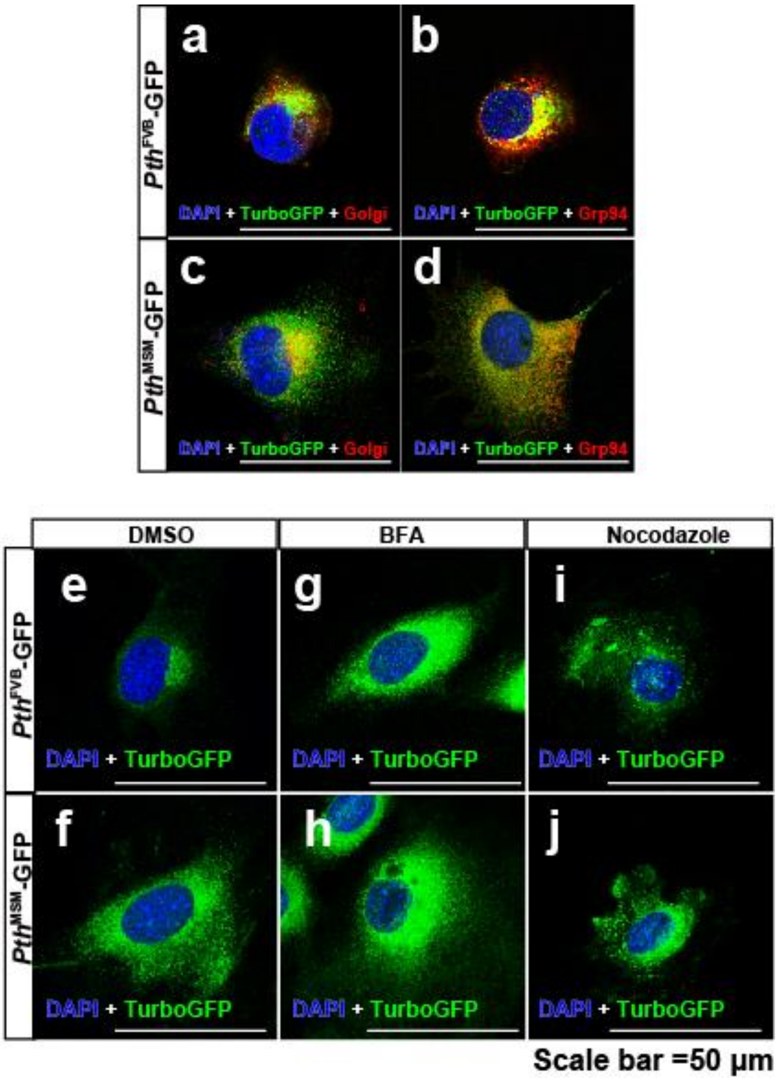

Supplementary Figure S5. Immunofluorescence staining of PTH-GFP in *Pth*^{FVB}-and *Pth*^{MSM}-transfected NIH-3T3 cells and treatment with protein transport inhibitors. **a)-d)** Immunofluorescence staining of PTH-GFP in *Pth*^{FVB}- and *Pth*^{MSM} -transfected NIH-3T3 cells. Transfected cells were stained with anti-turboGFP antibody (green), in combination with TR Ceramide as a Golgi marker **(a, c)** or Grp94 as an ER marker **(b, d)** (red). Cells were counterstained with DAPI (blue). **e)-l)** Immunofluorescence staining of PTH-GFP in DMSO (control), BFA (10 mM) and Nocodazole (10 mM) treated *Pth*^{FVB}- and *Pth*^{MSM}-transfected NIH-3T3 cells. Transfected cells were stained with anti-turboGFP antibody (green), and cells were counterstained with DAPI (blue). Scale bars, 50 µm.

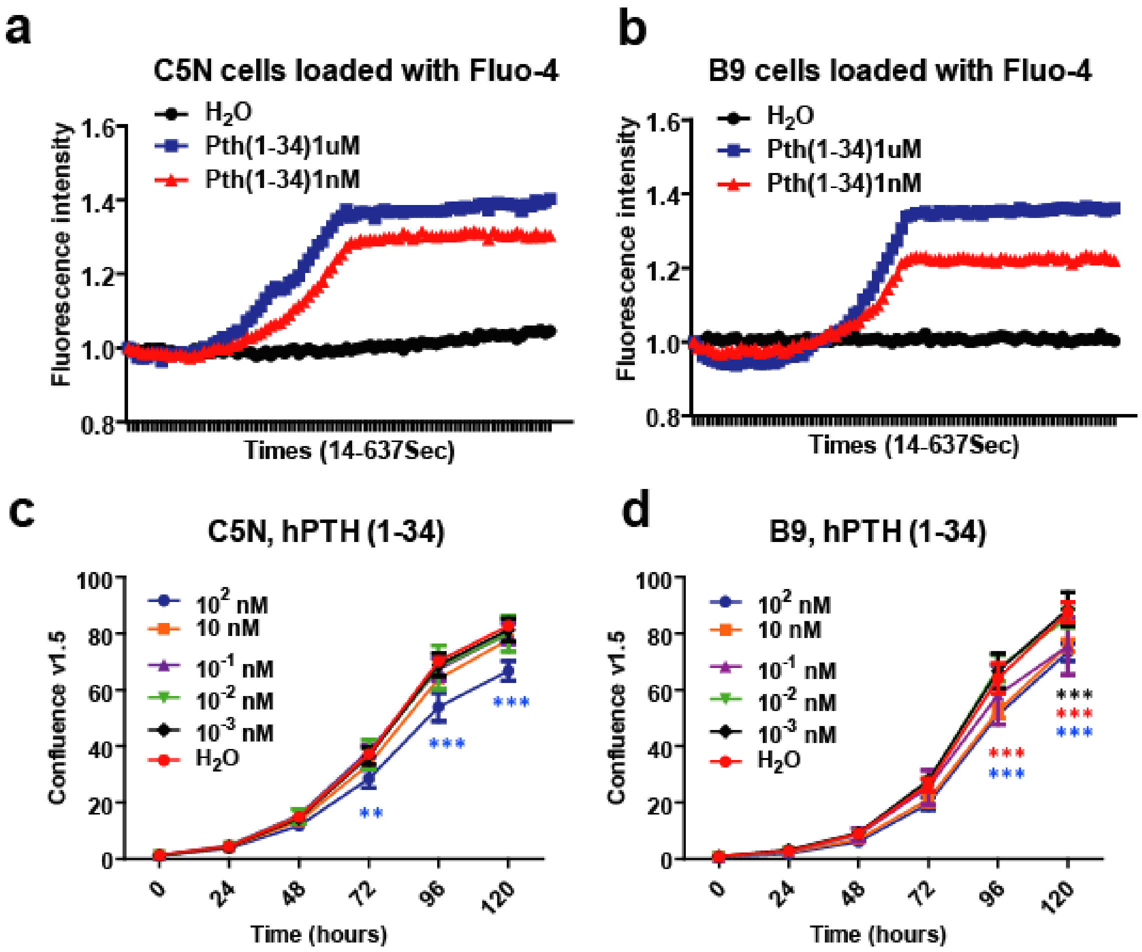

Supplementary Figure S6. A PTH agonist (hPTH(1-34)) increases intracellular calcium and suppresses cell proliferation in C5N and B9 cells. a), b) Intracellular calcium was monitored for 637 seconds after adding 1 nM and 1 μM of hPTH(1-34) to C5N **(a)** and B9 **(b)** cells. **c), d)** Cell proliferation assay after adding an agonist, hPTH(1-34). This agonist was added to $1{\times}10^3$ cells of C5N **(a, c)** or B9 **(b, d)** seeded on 96 well plates. **c), d)** Proliferation curves represent means of confluence values ± SEM of triplicates. The *P*-values were calculated by two-way ANOVA. Black asterisks indicate H_2O vs 10^{-1} nM. Red asterisks indicate H_2O vs 10 nM. Blue asterisks indicate H_2O vs 10^2 nM (***P < 0.001, **P < 0.01, *P < 0.05). Asterisks indicate significant differences. Error bars are standard deviation (SD).

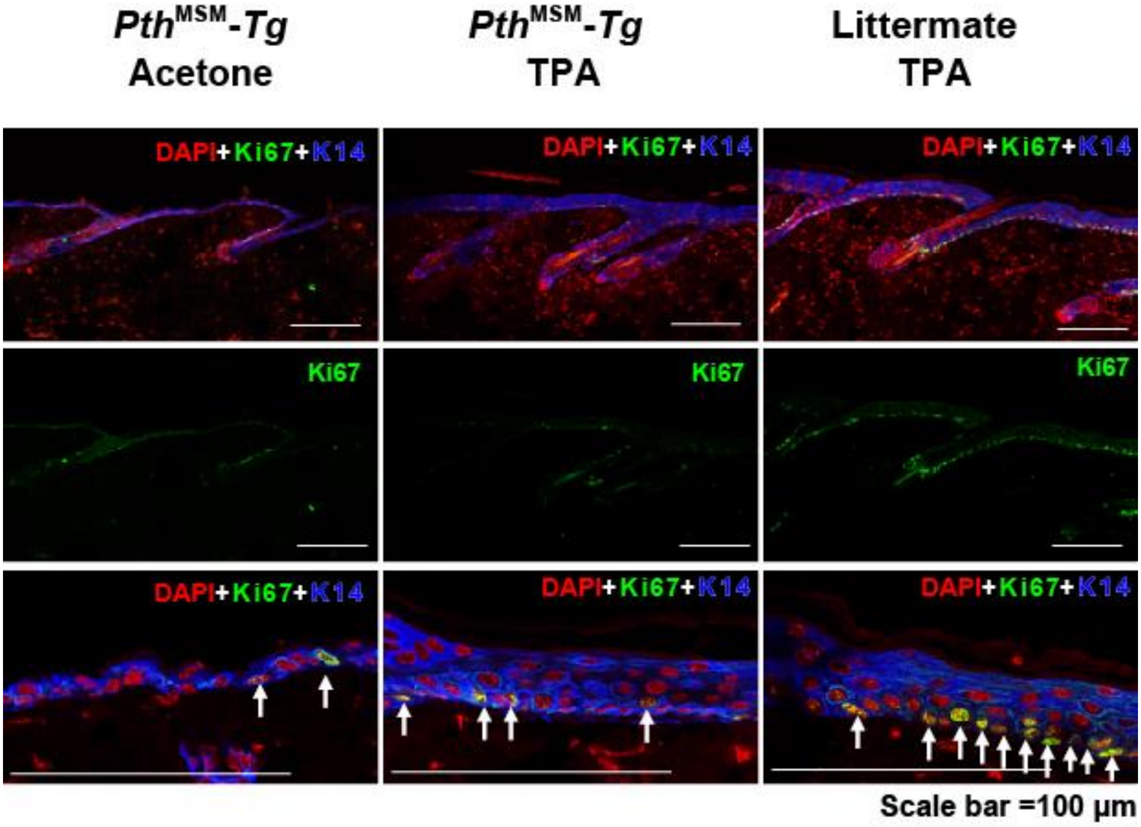

Supplementary Figure S7. PTH inhibits TPA-induced hyper-proliferation in the epidermis. *Pth*^{MSM} -*Tg* and their littermates were treated once with TPA (10 μg per mouse in 200 μl of acetone), and skin was collected 48 hours later. Acetone treated *Pth*^{MSM}-*Tg* mice were used as controls. Immunofluorescence staining of proliferative cells in the epidermis was by Anti-Ki-67 antibody (green) in combination with anti-K14 antibody (blue). Cell nuclei were counterstained with DAPI (red). White arrows indicate Ki-67-positive cells. Scale bars, 100 μm.

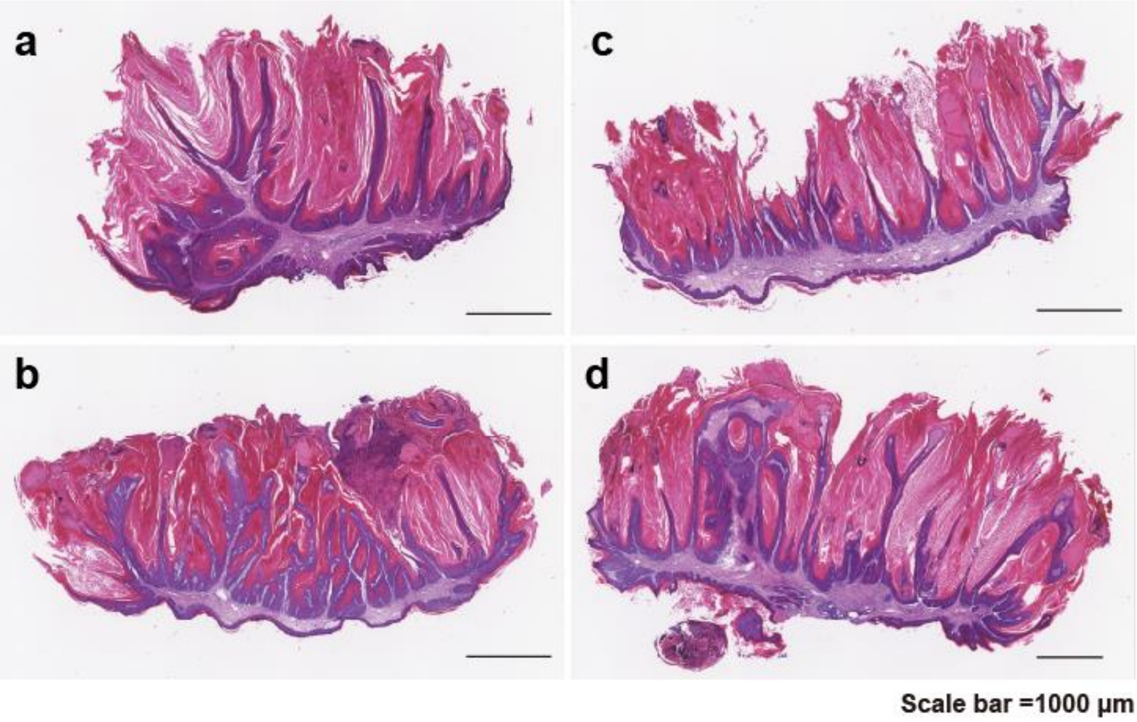

Supplementary Figure S8. Histological analysis of chemically induced papillomas from *Pth*^{MSM}*-Tg* and *Stmm1b* sub-congenic mice. **a)** HE staining of a papilloma from a *Pth*^{MSM}-*Tg* mouse in 20 weeks after DMBA treatment. **b)** HE staining of a papilloma from a wild type littermate of *Pth*^{MSM}-*Tg* mice in 20 weeks after DMBA treatment. **c)** HE staining of a papilloma from a *Stmm1b*$^{MSM/FVB}$ in 20 weeks after DMBA treatment. **d)** HE staining of a papilloma from a *Stmm1b*$^{FVB/FVB}$ mouse in 20 weeks after DMBA treatment. Scale bars, 1,000 µm.

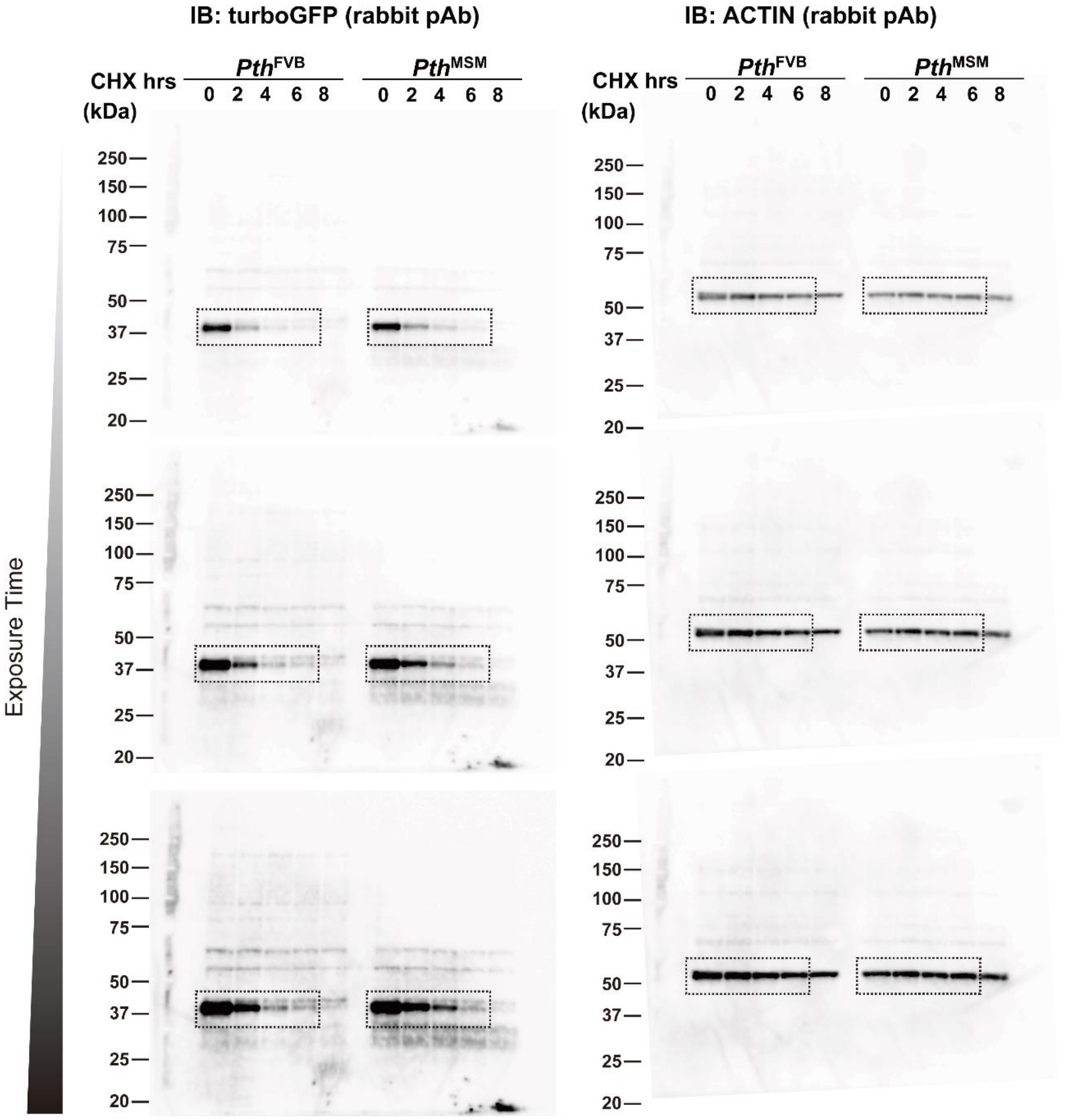

Supplementary Figure S9. The full-length blots for Figure 4g. Dashed line boxes indicate the cropped images used in Fig. 4g. The images with three different exposure times are shown for turbo GFP and ACTIN, respectively.

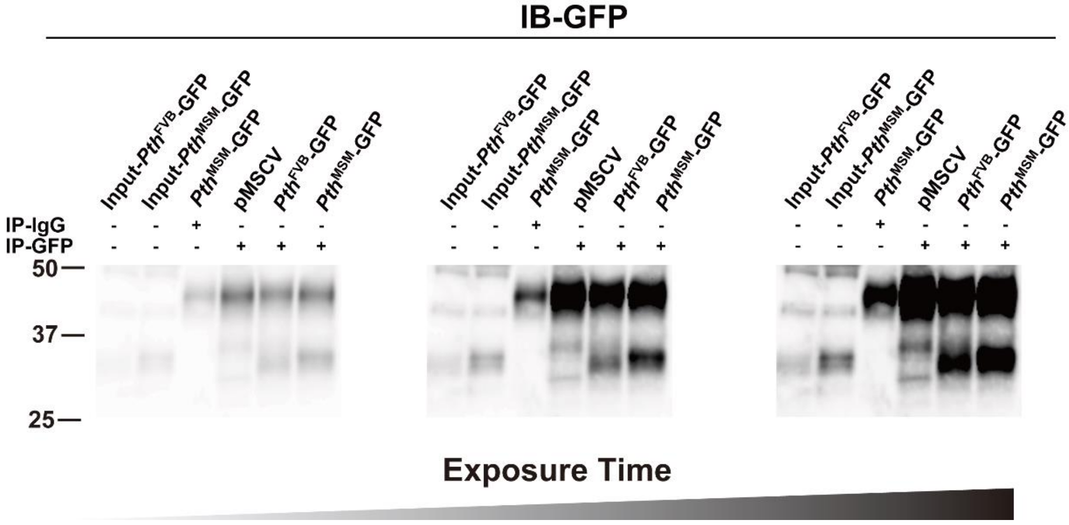

Supplementary Figure S10. The image used in Fig. 4i. The images with different exposure times are shown.

**Table S1.** Primers used for the genotyping of MSM-BAC clone				
**Gene Symbol**	**Primer Name**	**Primer Sequence (5' - 3')**	**Expected Product Size (bp)**	**Cycle conditions for PCR**
Arntl	Arntl-F	ACCAAGGATCAAGTAGTCCCAGTA	493	95 °C, 5 min, 1 cycle (94 °C, 30 s; 60 °C, 30 s; 72 °C, 30 s) 40 cycles
	Arntl-R	ATGGGGGACTTCTTTGTAGTGTAA		
Btbd10	Btbd10-F	CGTCGTACTCCTCCTTCTGC	583	
	Btbd10-R	ACCACCTCTTGACGTTCACC		
Pth	Pth-F3	GCAAACACCGTGGCTAAAGT	249	
	Pth-R1	CTCCTTCTTGGTGGGCTTCT		
047A16T7(BAC)	047AT7-F	CGTATTTGCGTTAAATGAAGGAG	490	
	047AT7-R	TCCATCTATGTCTGGGAGAAGAA		
047A16TJ(BAC)	047ATJ-F	GAAACCCTGTCTCGAAAAACTG	526	
	047ATJ-R	TGGACAGATCCCTATACCCACT		
466J23T7(BAC)	466JT7-F	GACATGGAAGCTAGAAAAGGACAT	176	
	466JT7-R	AAGCAACAATTTAAACCTGGAAAG		
466J23TJ(BAC)	466JTJ-F	ATGTGCTTCCTCTAGTTAGTGACG	245	
	466JTJ-R	GGACATCTCTATCCTTTTTCTCCA		
*Arntl**	Arntl-Ms-F	CAGTACTGGGGTGGGTAGGA	228	
	Arntl-Ms-R	TGCTTCAGAGACCTGCAAGA		
*Btbd10**	Btbd10-Ms-F	GAGGAAAGGCCCTTGGTCTA	151	
	Btbd10-Ms-R	CGGCAGGCAGAAAAGTAAAG		

*These are microsatellite markers. *Arntl* primers were designed around the (CA) repeat region in intron 3. The band size was 228 bp (FVB) and approximately 150 bp (MSM). *Btbd10* primers were designed for amplification of the (CA) region in intron 8. The band size was 151 bp (FVB) and approximately 140 bp (MSM).

**Table S2.** Primers used for real-time PCR analysis				
**Gene Symbol**	**Primer Name**	**PrimerSequence (5' - 3')**	**Expected Product Size (bp)**	**Reference**
Pth	Pth-F4	CTGCAGTCCAGTTCATCAGC	507	Liu Z et al., 2010
	Pth-R4	AAGCTTGAAAAGGTAGCAGCA		
Pthrp	Pthrp-F	CAGCCGAAATCAGAGCTACC	206	—
	Pthrp-R	CTCCTGTTCTCTGCGTTTCC		
Pth1r	Pthr1-F	GGACTCAGCCTTCCCCTTAG	220	—
	Pthr1-R	CTTCCTGCAACAATGGAGGT		
Lor	Lor-F	CCTGTGGGTTGTGGAAAGACC	117	Geng S *et al*., 2006
	Lor-R	AGAGCCTCCTCCAGATGAGC		
Flg	Flg-F	GTTTCCAAACACATGGATCAAAT	247	Hansmann B *et al*., 2012
	Flg-R	TTTGAATCTTGTTGGTGTCTGTG		
Krt10	Krt10-F	CGTACTGTTCAGGGTCTGGAG	65	Omori-Miyake M *et al*., 2013
	Krt10-R	GCTTCCAGCGATTGTTTCA		
Actb	F2-beta actin	ACCTCATGAAGATCCTGACC	195	—
	R2-beta actin	CGTTGCCAATAGTGATGACC		
